# Autocatalytic cores in the diluted regime: classification and properties

**DOI:** 10.1007/s00285-026-02357-7

**Published:** 2026-02-21

**Authors:** Praneet Nandan, Philippe Nghe, Jérémie Unterberger

**Affiliations:** 1 Laboratoire de Biophysique et Evolution, UMR CNRS-ESPCI 8231 Chimie Biologie Innovation, ESPCI Paris Université PSL, 10 Rue Vauquelin, 75005 Paris, France; 2 Laboratoire de Biologie Structurale de la Cellule (BIOC), CNRS, Ecole Polytechnique, Institut Polytechnique de Paris, 91120 Palaiseau, France; 3 Institut Elie Cartan, Université de Lorraine, B.P. 239, F – 54506 Vandœuvre-lès-Nancy Cedex, France

**Keywords:** open reaction network, autocatalysis, autocatalytic cores, Lyapunov exponent, growth rate, stationary state, equilibrium state, unistationarity, global attractor conjecture

## Abstract

Autocatalysis underlies the ability of chemical and biochemical systems to replicate. Autocatalysis was recently defined stoichiometrically for reaction networks; five types of minimal autocatalytic networks, termed autocatalytic cores were identified. A necessary and sufficient stoichiometric criterion was later established for dynamical autocatalysis in diluted regimes, ensuring a positive growth rate of autocatalytic species starting from infinitesimal concentrations, given that degradation rates are sufficiently low. Here, we show that minimal autocatalytic networks in the dynamical sense, in the diluted regime, follow the same classification as autocatalytic cores in the stoichiometric sense. We further prove the uniqueness of the stationary regimes of autocatalytic cores, with and without degradation, for all types, except types II with three catalytic loops or more, for which the question remains open. These results indicate that the stationary point is robust under perturbation at low concentrations. More complex behaviours require additional non-linear couplings.

**Supplementary Information:**

The online version contains supplementary material available at 10.1007/s00285-026-02357-7.

## Introduction

The ability of living systems to reproduce themselves is based on the chemical mechanism of autocatalysis. In extant biology, autocatalysis arises via DNA replication, coupled to a highly complex network of biochemical reactions that ensure metabolism, gene expression, membrane production, etc. However, autocatalysis must have been present in the early stages of life, possibly even during so-called chemical evolution (Kauffman 1986; Lancet et al. 2018; Oparin 1952), in a more rudimentary form. Indeed, self-reproduction is a necessary property for Darwinian evolution, which was itself required for elaborate biochemical enzymes to appear.

Only recently, autocatalysis has been formally defined at the level of reaction networks, where multiple species altogether participate in the process of self-amplification (Andersen et al. 2021; Blokhuis et al. 2020). Blokhuis et al. (2020) defined general stoichiometric conditions for autocatalysis in reversible reaction networks. They deduced five classes of minimal network topologies, called autocatalytic cores, meaning that any autocatalytic network must contain an autocatalytic core as a subnetwork. These conditions allow algorithms to systematically detect autocatalysis in large reaction networks, such as metabolisms (Arya et al. 2022; Peng et al. 2022).

Autocatalysis must also fulfill dynamical conditions, where every autocatalytic species increases in concentration over time (Kamimura et al. 2024; Lin et al. 2020; Srinivas et al. 2023, 2024). Indeed, reactions that degrade autocatalysts into side products may lead to the extinction of the overall network. Blokhuis et al. further provided viability conditions, based on stochastic processes, where dynamical viability is computed on a case-by-case basis using branching processes, assuming that autocatalysts are initially absent, or present in a few copy numbers. Reference (Unterberger and Nghe 2022) proved a general stoichiometric criterion for dynamical autocatalysis in the diluted regime, given small enough degradation reactions. The notion of diluted regime, used in the present work and formally defined in the next section, is that of infinitesimal initial concentrations. This regime is realized at the onset of autocatalytic amplification. Thus it characterizes the ability of autocatalysis to start spontaneously, or from a perturbation wherein chemical species are transiently injected into the reaction vessel (e.g. a meteorite landing). In this regime, the probability of two species to meet becomes negligible compared to one-to-one and one-to-many reactions. The general, rate-independent criterion for dynamical autocatalysis then boils down to having a strongly connected chemical network containing at least a one-to-many reaction.

However, the relationship between stoichiometry and dynamics of autocatalytic networks remains an essentially open question. The first question is that of minimal autocatalytic cores in the dynamical sense. In the diluted regime, dynamical cores must contain autocatalytic cores. However, the shift to the diluted regime can cause some reactions to become irreversible. Stoichiometric autocatalysis has not yet been classified for reaction networks comprising irreversible reactions. In the present work, we enumerate minimal structures obeying diluted dynamical autocatalysis, and find that they are a subset of stoichiometric autocatalytic cores found for reversible reaction networks. A second question is that of multistationarity. While chemical evolution requires multiple growth states (Ameta et al. 2022; Peng et al. 2020) and autocatalytic cores are a possible candidate to trigger dynamical instabilities (Vassena and Stadler 2024), it remains unknown whether autocatalysis is prone to complex dynamics such as multistationarity. Here, we show that autocatalytic cores have a single stationary state, with or without degradation reactions. This result builds on deficiency theory augmented by an analysis of the behaviour of solutions when continuously varying degradation rates.

Here is an outline of the article. In section 2, we recall former results on stoichiometric and dynamical autocatalysis and provide useful definitions. The classification theorem for dynamical autocatalytic cores is stated in section 3. In Section 4 we state and give the proof for the uniqueness of the stationary states for these cores in the absence of degradation. Section 5 contains our main results for the stationary states of these cores with degradation, along with Theorem 5.1, which plays a vital part in the proof. We discuss our results in Section 6 and some technical details and definitions are postponed to Section 7. The proof of the classification theorem in Section 3, along with the details of the calculations in Section 4 and 5 are in the Supplementary Information.

## Background results and definitions

### Autocatalytic cores

We state definitions used for the stoichiometric classification of minimal autocatalytic networks, called cores, as introduced in reference (Blokhuis et al. 2020). A reaction network is defined by its graph $G=(\mathcal {X},\mathcal {R})$, with $\mathcal {X}=\{x_1,\ldots ,x_n\}$ the set of species, and $\mathcal {R}$ = set of reactions. All reactions are assumed to be **autonomous**, meaning that they have at least one reactant and one product, and **non-ambiguous**, meaning that the same species is either a reactant or a product, but never both (Blokhuis et al. 2020).

Consequently, reactions are of the general form

2.1 $$\begin{aligned} &  R \text { (reversible) }\, : \qquad s_1 x_1+\ldots +s_k x_k \leftrightarrows s'_1 x'_1+\ldots +s'_{k'} x'_{k'} \end{aligned}$$

2.2 $$\begin{aligned} &  R_i \text { (irreversible) }\, : \qquad s_1 x_1+\ldots +s_k x_k \rightarrow s'_1 x'_1+\ldots +s'_{k'} x'_{k'} \end{aligned}$$

with $k,k'\ge 1$, all species $x_1,\ldots ,x_k,x'_1,\ldots ,x'_{k'}$ distinct, and $s_1,\ldots ,s_k; s'_1,\ldots ,s'_{k'}\in \mathbb {N}_{>0}$ integer stoichiometric coefficients. For irreversible reaction $R_i$ (2.2), we also define their reverse:

2.3 $$\begin{aligned} \bar{R_i} \text { (irreversible) }\, : \qquad s'_1 x'_1+\ldots +s'_{k'} x'_{k'}\rightarrow s_1 x_1+\ldots +s_k x_k \end{aligned}$$

where the reactants and the products exchange places. Note that for reversible reactions, $\bar{R}$ and *R* are equivalent.

Choosing an ordering $\mathcal {R}=\{R^{(1)},\ldots ,R^{(m)}\}$ of $\mathcal {R}$, the columns $$\left( \begin{array}{c} -s^{(j)}_1 \\ \vdots \\ -s^{(j)}_k \\ \hline (s^{(j)})'_1 \\ \vdots \\ (s^{(j)})'_{k'} \end{array}\right) $$ of the stoichiometric coefficients of $R^{(1)},\ldots , R^{(j)},\ldots , R^{(m)}$ form an $n\times m$ matrix $\mathbb S$ called *Stoichiometric matrix*.

*Non-degenerate networks.* A network is *non-degenerate* if and only if rk(${\mathbb S})=|\mathcal {X}|$.

**Note:** Our non-degeneracy assumption excludes conserved quantities. This is a choice we make keeping in mind that autocatalytic cores do not have any mass-like conservation law.

*Mass-action dynamics.* Choose direct, resp. reverse non-negative rates $k_+^{(j)}$, resp. $k_-^{(j)}$ (for irreversible reaction, one of the rates is 0) for the reaction

2.4 $$\begin{aligned} R^{(j)}: s_{j,1} x_{i_{j,1}}+\ldots +s_{j,k_j} x_{i_{j,k_j}} \overset{k^{(j)}_+}{ \underset{k^{(j)}_-}{\rightleftarrows }} s'_{j,1} x_{i'_{j,1}}+\ldots +s'_{j,k'_j} x_{i'_{j,k'_j}} \end{aligned}$$

Let

2.5 $$\begin{aligned} C:= ([x_i])_{x_i\in \mathcal {X}} \end{aligned}$$

be the vector of species concentrations. *C* is an element of $\mathbb {R}^{\mathcal {X}}$, spanned by the canonical basis $(e_{\ell })$ defined by $(e_{\ell })_x=\delta _{\ell ,x}$. Then the associated mass-action dynamics can be written in terms of the stoichiometric matrix as

2.6 $$\begin{aligned} \frac{dC}{dt} = {\mathbb S} J(C), \end{aligned}$$

where the **current** $J=J(C)$ is the vector with components

2.7 $$\begin{aligned} J^{(j)}(C)= k^{(j)}_+ \prod _{\ell =1}^{k_j} ([x_{i_{j,\ell }}])^{s_{j,\ell }} - k^{(j)}_- \prod _{\ell '=1}^{k'_j} ([x_{i'_{j,\ell '}}])^{s'_{j,\ell '}} \qquad (j=1,\ldots ,m). \end{aligned}$$

Alternatively, treating separately direct and reverse reactions, $\frac{dC}{dt}$ may be written in terms of **flows**; namely, the direct reaction $R^{(j)}$ is characterized by the two stoichiometric vectors $(y_{R^{(j)}},y'_{R^{(j)}})$, with

2.8 $$\begin{aligned} y_{R^{(j)}}:= s_{j,1} e_{i_{j,1}} + \ldots + s_{j,k_j} e_{i_{j,k_j}}, \qquad y'_{R^{(j)}}:= s'_{j,1} e_{i'_{j,1}} + \ldots + s'_{j,k_j} e_{i'_{j,k_j}} \end{aligned}$$

called respectively **reactant composition** and **product composition**, and by the direct flow

2.9 $$\begin{aligned} \phi _{y_{R^{(j)}} \rightarrow y'_{R^{(j)}}} (C) := k^{(j)}_+ \prod _{\ell =1}^{k_j} ([x_{i_{j,\ell }}])^{s_{j,\ell }} \end{aligned}$$

whereas the associated reverse reaction is characterized by the permuted pair $(y'_{R^{(j)}},y_{R^{(j)}})$ and by the reverse flow

2.10 $$\begin{aligned} \phi _{y'_{R^{(j)}} \rightarrow y_{R^{(j)}}} (C) := k^{(j)}_- \prod _{\ell '=1}^{k'_j} ([x_{i'_{j,\ell '}}])^{s'_{j,\ell }} \end{aligned}$$

Note that currents

2.11 $$\begin{aligned} J^{(j)}(C) = \phi _{y_{R^{(j)}} \rightarrow y'_{R^{(j)}}} (C) - \phi _{y'_{R^{(j)}} \rightarrow y_{R^{(j)}}} (C) \end{aligned}$$

are obtained by ’antisymmetrizing’ flows. Combining the above equations, one gets

2.12 $$\begin{aligned} \frac{dC}{dt} = \sum _{y\rightarrow y'} \phi _{y\rightarrow y'}(C) (y'-y) \end{aligned}$$

where the sum ranges over all direct or reverse reactions *R* characterized by their stoichiometric pair $(y,y')=(y_R,y'_R)$.

*Positive vectors.* Let $v\in \mathbb {R}^p$ $(p=n$ or *m*) be a vector. We say that *v* is positive $(v>0)$ if all its components are positive, i.e., $v_i>0$ for all *i*.

We assume mass action kinetics, as except for the case of bound or precipitating species, all reactions can be broken down into elementary steps where these kinetics hold. Furthermore, as we prove our results without any assumptions on the rate constants, they are not dependent on the change in reaction rates resulting from this decomposition into elementary steps. In an evolutionary context without reactions involving ions, it is an adequate assumption for studying autocatalytic cycles.

#### Definition 2.1

*(stoichiometrically autocatalytic network)* (see Blokhuis et al. (2020)) An autonomous and non-ambiguous reaction network $G=(\mathcal {X},\mathcal {R})$ is stoichiometrically autocatalytic if and only if there exists a positive column vector $$c=\left( \begin{array}{c} c^{(1)} \\ \vdots \\ c^{(m)} \end{array}\right) $$ such that ${\mathbb S}c>0$.

In reference (Unterberger and Nghe 2022), the distinction is made between *stoichiometrical autocatalysis* and *dynamical autocatalysis*. While the former is a condition where the stoichiometrical balance for all autocatalytic species is positive, Dynamical autocatalysis is the property for the system to grow exponentially from very low concentration (with a strictly positive Lyapunov exponent).

#### Definition 2.2

*(dynamically autocatalytic network)* (see Unterberger and Nghe (2022)) A reaction network $G=(\mathcal {X},\mathcal {R})$ is dynamically autocatalytic if and only if the underlying (mass action) dynamical system has a strictly positive largest Lyapunov exponent with a corresponding non-negative Lyapunov eigenvector.

#### Definition 2.3

*(restricted network)* Let $G=(\mathcal {X},\mathcal {R})$ be a network, and $\mathcal {X}'\subset \mathcal {X}$. Then

2.13 $$\begin{aligned} G\Big |_{\mathcal {X}'}:=(\mathcal {X}',\mathcal {R}\Big |_{\mathcal {X}'}) \end{aligned}$$

called *restriction of G* to $\mathcal {X}'$, is the subnetwork of *G* obtained by restricting the set of species to $\mathcal {X}'$ and **chemostatting** species not in $\mathcal {X}'$, by

1. removing reactions having either all reactants or all products (or both) in $\mathcal {X}\setminus \mathcal {X}'$ (as non-autonomous after chemostatting);
2. in all other reactions, replacing all stoichiometric coefficients of species in $\mathcal {X}\setminus \mathcal {X}'$ by 0.

Alternatively, one may also restrict the reaction set $\mathcal {R}$ to $\mathcal {R}'\subset \mathcal {R}$; the result is simply denoted $(\mathcal {X}, \mathcal {R}')$. The two restriction operations can be composed in either direction.

#### Definition 2.4

*(minimality, autocatalytic cores)* An autocatalytic network (stoichiometric or dynamic) $G=(\mathcal {X},\mathcal {R})$ is minimal if

2.14 $$\begin{aligned} \Big ( \mathcal {R}'\subseteq \mathcal {R}, \mathcal {X}'\subseteq \mathcal {X}, (\mathcal {X},\mathcal {R}')\Big |_{\mathcal {X}'}\ {\text {autocatalytic}} \Big ) \Longrightarrow \Big ( \mathcal {R}'= \mathcal {R}, \mathcal {X}'=\mathcal {X} \Big ). \end{aligned}$$

A minimal autocatalytic network is called a stoichiometric/dynamic (resp.) *autocatalytic core*.

#### Definition 2.5

*(Reversible extension)* The *reversible extension* of a network $G=(\mathcal {X}, \mathcal {R})$ is the network $\overleftrightarrow {G}=(\mathcal {X},\overleftrightarrow \mathcal {R})$ obtained from *G* by making reversible *all* reactions in $\mathcal {R}$, i.e.2.152.16(See reactions ((2.1)) and ((2.2))

### Diluted regime

Dynamical autocatalysis — the ability of autocatalytic reaction networks to kinetically (and exponentially) amplify their species — has been studied in the context of the *diluted regime* (Unterberger and Nghe 2022), i.e. infinitesimally low concentrations. It is possible to translate the notion of dilution in terms of the topology of *G* because when species concentrations are small enough, reactions involving multiple reactants become negligible as compared to those comprising a single reactant. In other terms, many-to-one or many-to-many reactions are negligible as compared to one-to-one or one-to-many reactions, following the definitions:

- one-to-one (1–1) reactions have stoichiometry $(-1;1)$, i.e. they are of the form $x\rightarrow x'$ or $x\leftrightarrows x'$, with $x\not =x'\in \mathcal {X}$; They can be reversible or irreversible.
- one-to-many reactions are irreversible and have stoichiometry $(-1;s'_1,\ldots ,s'_{m'})$ with $s'_1,\ldots ,s'_{m'}=1,2,\ldots$, $\sum _i s'_i\ge 2$, i.e. they are of the form $x\rightarrow s'_1 x'_1 +\ldots + s'_{m'} x_{m'}$.
- many-to-one reactions are also irreversible and are those obtained by reversing one-to-many reactions, i.e. $s'_1 x'_1 +\ldots + s'_{m'} x_{m'} \rightarrow x$.
- many-to-many reactions can be both reversible or irreversible and have stoichiometry $(-s'_1,\ldots ,-s'_{m'}; s_1,\ldots ,s_m)$ with $\sum _i s'_i\ge 2$ and $\sum _i s_i\ge 2$.
  Stoichiometric coefficients $s'_i,s_i$ play little role in the discussion, therefore we more simply refer to $m,m'$ alone:
- a *simple* reaction has $m'=m=1$, i.e. it is of the form $s_1 x_1\rightarrow or \leftrightarrows s'_1 x'_1$, with $s_1,s'_1=1,2,\ldots$ arbitrary;
- a *multiple product* reaction has $m'\ge 2$, i.e. it is of the form $s_1 x_1+\ldots +s_m x_m \rightarrow s'_1 x'_1+\ldots +s'_{m'} x'_{m'}$ with $m'\ge 2$.

#### Definition 2.6

*(diluted reaction networks)* A reaction network is *diluted* if it contains only simple reactions, and possibly some irreversible one-to-many reactions (i.e. multiple product reaction where m=1, single reactant).

Reversible extensions of diluted autocatalytic cores will be used in section 4, where we show that they possess a single unique stationary state.

### (Top) property

*Classes.* Consider the Stoichiometric matrix $\mathbb S$. Now consider the directed graph $G({\mathbb S})$ obtained by connecting reactants to products participating in the same (reversible or irreversible) reaction. *G* is in fact the graph of the *split reactions*:

*Split reactions.* The arrow $x \rightarrow x'$ is a split reaction iff $\mathcal {R}$ contains a reaction $x\rightarrow s'x'+s'_2 x'_2+\ldots + s'_{k'}x'_{k'}$ or $x\leftrightarrows s'x'+s'_2 x'_2+\ldots + s'_{k'}x'_{k'}$, i.e. if it is an edge of $G({\mathbb S})$.

We colloquially refer to *G* as the *split graph*, as it consists in splitting one-to-many reactions into as many edges as products. The strongly connected components of $G({\mathbb S})$ are called *classes*.

*Class graph.* Contracting all species within a given class to a point results in a directed acyclic graph (called: *class graph*). This graph defines a partial order wherein $\mathcal {C}$ is *upstream* from $\mathcal {C}'$, denoted $\mathcal {C}\prec \mathcal {C}'$, if there exists a split reaction $x\rightarrow x'$ with $x\in \mathcal {C},x'\in \mathcal {C}'$.

*Minimal classes.* $\mathcal {C}$ have the property that no class is located upstream from them, i.e. there is no split reaction $x'\rightarrow x$ with $x'\not \in \mathcal {C},x\in \mathcal {C}$.

*An example.* Take the reaction network with the reactions

2.17 $$\begin{aligned} &  x_i \rightarrow x_j + x_k \nonumber \\ &  x_i \rightleftarrows x_l \nonumber \\ &  x_k\rightleftarrows x_m \end{aligned}$$

The split graph is 
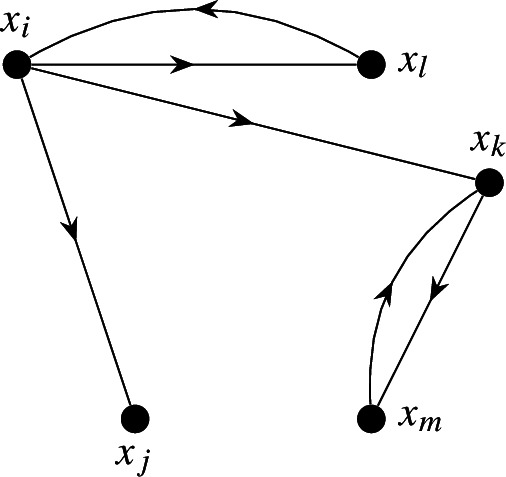
 The corresponding classes are $\mathcal {C}_i=\{x_i,x_l\},\mathcal {C}_j=\{x_j\},\mathcal {C}_k=\{x_k,x_m\}$, and the Class graph is shown below. Note that $\mathcal {C}_i$ is the only minimal class, and is "upstream" of $\mathcal {C}_j,\mathcal {C}_k$
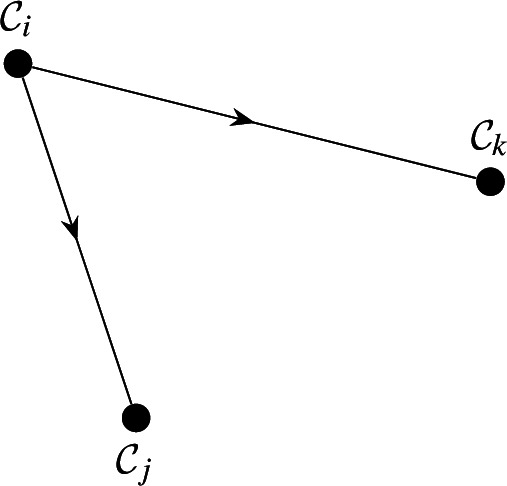


*Restricted reactions.* Let $\mathcal {C}$ be a class. If $R \, :\, x\rightarrow s'_1 x'_1+\cdots + s'_{k'} x'_{k'}$ is a reaction such that $x\in \mathcal {C}$, and $\{i=1,\ldots ,k' \ |\ x'_i\in \mathcal {C}\}=\{1,\ldots ,j'\}$ for some $j'\ge 1$, then the $\mathcal {C}$**-truncated reaction** is

2.18 $$\begin{aligned} R\Big |_{\mathcal {C}}\, :\, x\rightarrow s'_1 x'_1+\cdots + s'_{j'} x'_{j'}. \end{aligned}$$

$R\Big |_{\mathcal {C}}$ is obtained by chemostatting (keeping constant the concentration of) species, which do not belong to $\mathcal C$.

#### Definition 2.7

*(Irreducible Network)* A reaction network is irreducible if and only if the split graph G is strongly connected.

We now recall from (Unterberger and Nghe 2022) the (Top) property (signifying the topological nature of the property) and result, which stoichiometrically characterize dynamical autocatalytic networks in the diluted regime:

#### Definition 2.8

*((Top) property for autocatalysis)* A diluted reaction network *G* satisfies (Top) if all minimal classes of the adjacency graph $G({\mathbb S})$ contain at least one internal one-to-many reaction.

#### Proposition 2.9

A diluted network $G=(\mathcal {X},\mathcal {R})$ is (stoichiometrically) autocatalytic if and only if it satisfies (Top). Also if it satisfies (Top), it is dynamically autocatalytic.

#### Proof

See Theorem 3.1 in Unterberger and Nghe (2022) $\square$

#### Remark

An irreducible, diluted network $G=(\mathcal {X},\mathcal {R})$ has $|\mathcal {R}|\ge |\mathcal {X}|$ (for each $x\in \mathcal {X}$, there must exist at least one reaction $x\rightarrow \cdots$). Therefore, such a network is *non-degenerate* (i.e. rk$({\mathbb S})\ge |\mathcal {X}|$) if and only if ${\mathbb S}$ is *full-rank*, in which case rk$({\mathbb S})= |\mathcal {X}|$.

## Classification of dynamical autocatalytic cores

Stoichiometric autocatalytic cores were classified in Blokhuis et al. (2020). The dilute regime assumption (i.e. dismissing many-to-one or many-to-many reactions) involves a shift from a completely reversible network as considered in Blokhuis et al. (2020) to a network that can have irreversible reactions. In this regime, using Proposition 2.9, it can again be proved graph-theoretically that we get only five types of cores if we consider dilute dynamical autocatalytic cores. This proof is extensive and thus postponed to S1 in Supplementary Information. Here we just state the result, and then discuss how it relates to the one obtained in Blokhuis et al. (2020).

### Definition 3.1

*(diluted autocatalytic core)* An open reaction network $G=(\mathcal {X},\mathcal {R})$ is a *diluted autocatalytic core* if and only if it satisfies the (Top) property (Definition 2.8), is minimal, and is composed uniquely of one-to-one or one-to-many reactions (i.e. diluted).

### Theorem 3.1

Let $G=(\mathcal {X},\mathcal {R})$ be a diluted dynamical autocatalytic core. Then *G* is of one of the following types (generally speaking, possible products of one-to-many reactions are indicated by a bold line, see details below):

(i) Type I: all species lie along a cycle (see Definition 7.1 in Appendix) $\mathcal {C}=\{x_1\rightarrow x_2 \rightarrow \ldots \rightarrow x_n\rightarrow x_{n+1}=x_1\}$, all reactions are simple reactions along $\mathcal C$ in the same orientation, at least one of them is one-to-many, 
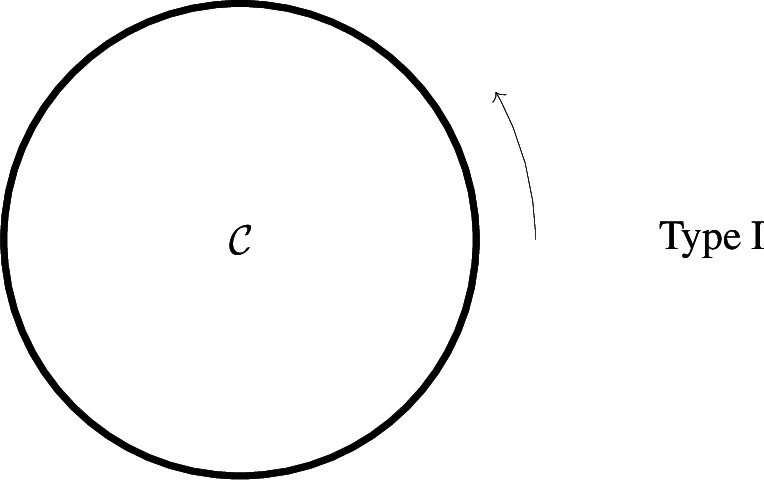
ii) Type $II_{\ell }$ ($\ell \ge 1$): all species lie along a cycle $\mathcal {C}=\{x_1\rightarrow x_2\rightarrow \cdots \rightarrow x_n\rightarrow x_1\}$, back-branches $i_j\rightarrow \sigma _j$, $i=1,\ldots ,\ell$ $(1=i_1\le \sigma _2<i_2\le \sigma _3<i_3\le \ldots \le \sigma _1<n+1)$, encoding split reactions originated from the multiple reactions $x_{i_j}\rightarrow s_{i_j} x_{i_j+1} + x_{\sigma _j}$, span disjoint arks along $\mathcal C$, 
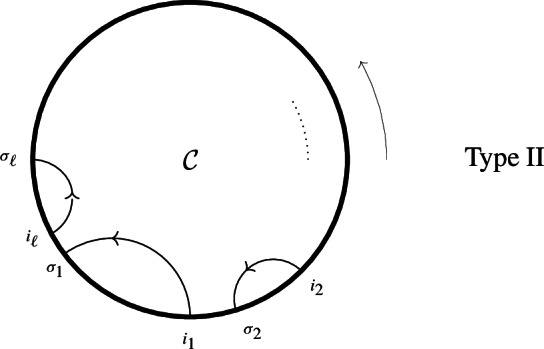
 Note that split reactions along $\mathcal {C}$ have arbitrary stoichiometry $x_i\rightarrow s_i x_{i+1}$ as in Type I, whereas back-branches have Stoichiometric coefficient 1.
(iii) Type III, all species lie either on first cycle $\mathcal C$ or on an ear (see Defintion 7.2) $\mathcal {O}= \mathcal {C}'\setminus (\mathcal {C}\cap \mathcal {C}')$ added to $\mathcal {C}$; $\mathcal {C}'\cap \mathcal {C}= \overrightarrow{(uv)}$ (possibly, $u=v$); reactions are simple reactions along cycles, plus a multiple reaction $v\rightarrow x+x'$ with reactant *v* and products $x\in \mathcal {C}\setminus (\mathcal {C}\cap \mathcal {C}')$, $x'\in \mathcal {C}' \setminus (\mathcal {C}\cap \mathcal {C}')$, 
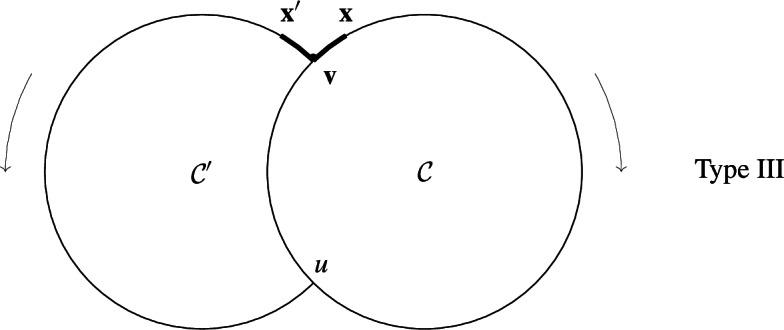

### Remark

By hypothesis, $x,x'\not =u$. However, if $x=u$ or $x'=u$, then Type III degenerates to Type II. If both $x=u$ and $x'=u$, then it further degenerates to Type I.

(iv) Type IV, same as Type III, but with an extra multiple reaction $w\rightarrow u+x'$ with reactant $w\in \mathcal {C}\setminus (\mathcal {C}\cap \mathcal {C}')$ (the case $x=w$ is not excluded) and products *u* and $x'$, 
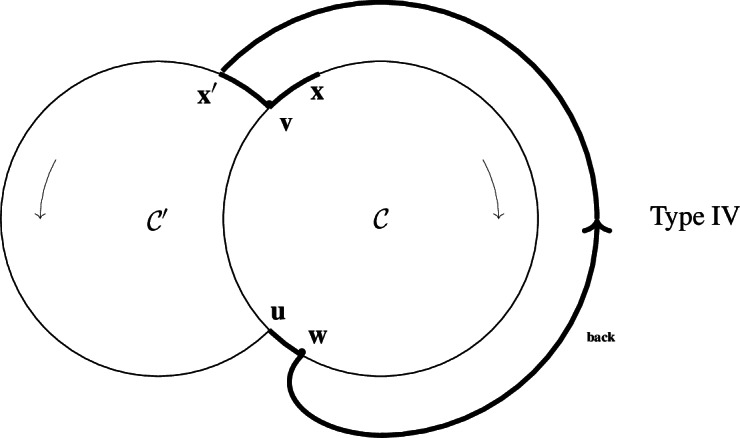
 The mention ’back’ on the edge $w\rightarrow x'$ refers to the *supplementary back-branch* $w\rightarrow x'$.
(v) Type V, same as Type IV with an extra multiple reaction $w'\rightarrow u+x$ with reactant $w'\in \mathcal {C}'\setminus (\mathcal {C}\cap \mathcal {C}')$ (the case $w'=x'$ is not excluded) and products *u* and *x*, 
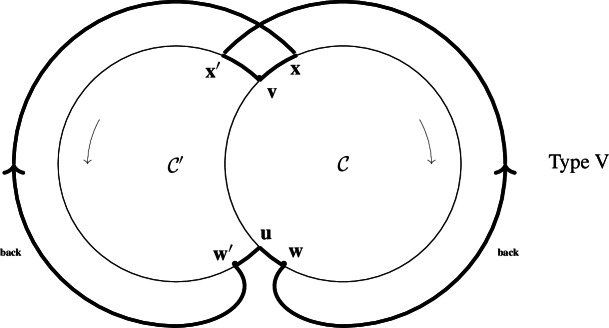
 The two mentions ’back’ refer to the supplementary back-branches $w\rightarrow x'$ and $w'\rightarrow x$.

Furthermore, all simple reactions in Types III,IV,V have stoichiometric coefficients 1.

In particular, *G* is always irreducible.

The more complete classification obtained in Blokhuis et al. (2020) without the dilute regime assumption is almost exactly the same (definition of Types I-V is copied from Blokhuis et al. (2020), see Suppl. Information, Fig. S1, with *u*, *v*, see Types III-IV-V, playing the same role to help identification). The difference comes from the fact that reactions of the type $sx\rightarrow s'_1 x'_1+\ldots +s'_{k'} x'_{k'}$ with $s\ge 2$ arise in general. Graphs are topologically the same, but minimality is reflected in supplementary vanishing, resp. non-vanishing conditions on the determinant of the stoichiometric matrix restricted to sub-cycles, the so-called weight-symmetric, resp. weight-asymmetric condition in Theorem 1 of Blokhuis et al. (2020). It is proved early on in that paper (Proposition 2) that reactions with several different reactants are not found in autocatalytic cores.

## Uniqueness of stationary states (without degradation)

We prove in this, and the next section, the uniqueness of stationary states for the reversible extensions of the diluted dynamical autocatalytic cores, which from now on we refer to as ’diluted autocatalytic cores’. This section covers the proof in the absence of degradation rates. We give an argument based on deficiency theory (Feinberg 1987, 1995, 2019). Further in Appendix 7.3, we give the explicit calculation of the Lyapunov function for the asymptotic stability, and the Supplementary information (section S2) contains the explicit form of the stationary state for each type of autocatalytic core, computed in terms of the rate constants.

In Subsection 4.2, we note how our work connects to the *Global Attractor Conjecture* (Horn 1974), and we show that for the diluted autocatalytic cores, the conjecture holds true.

The extension to cores with degradation, on the other hand, relies on tracking the solutions with changing degradation rates and inspecting degenerate stationary states, which is covered in the next section.

Recall from (2.6) that the dynamics is expressed by the equation $\frac{dC}{dt}= {\mathbb S}J(C)$.

*Stationary state.* A concentration vector *C* is a stationary state if and only if ${\mathbb S} J(C)=0$.

*Equilibrium states.* We say that a concentration vector *C* is an **equilibrium state** if all currents $J^{(j)}(C)$, $j=1,\ldots ,m$ vanish. It follows immediately from (2.6) that equilibrium states are stationary states. The reverse is not true in general.

### Proposition 4.1

For diluted autocatalytic cores, stationary states are equilibrium states.

### Proof

It was proved in Blokhuis et al. (2020) that all autocatalytic cores have an invertible stoichiometric matrix $\mathbb S$. Thus, specifically for autocatalytic cores, the stationary condition ${\mathbb S}J(C)=0$ implies that $J(C)=0$; in other words, stationary states are equilibrium states. $\square$

Open reaction networks equipped with mass-action rates do not always have a unique stationary state; they may have none or several, or also admit periodic orbits. Diluted autocatalytic cores are specific in this respect. As the concentrations of the species which are part of the core increase, the reverse reactions of the irreversible reactions in the core start to affect the dynamics of the system. Thus to be precise, we consider the reversible extension of a diluted autocatalytic core $\overleftrightarrow {G}=(\mathcal {X},\overleftrightarrow \mathcal {R})$, and choose direct, resp. reverse rates $k_+^{R}$, resp. $k_-^{R}$ for all (direct) reactions $R\in \mathcal {R}$. Then the following result holds:

### Theorem 4.1

(uniqueness of stationary states) Let $G=(\mathcal {X},\mathcal {R})$ be a diluted autocatalytic core, $\overleftrightarrow {G} = (\mathcal {X},\overleftrightarrow \mathcal {R})$ its reversible extension (see Definition 2.6), and $(k_+^R)_{R\in \mathcal {R}}$, resp. $(k_-^R)_{R\in \mathcal {R}}$ be arbitrary, positive direct, resp. reverse reaction rates. Then the corresponding reaction network admits a unique positive stationary state $C_{stat}$, which is an equilibrium state. Furthermore, for any initial concentration vector $C_0>0$, the solution of the dynamical system $\frac{dC}{dt}={\mathbb S}J(C)$, giving the time evolution of the concentration vector, converges to $C_{stat}$ as $t\rightarrow \infty$.

The second part of the theorem concerning the notion of *Persistence* (as defined in Feinberg (1989)) is addressed in Subsection 4.2.

Let us first review some definitions. Recall that $(e_x)_{x\in \mathcal {X}}$ is the standard basis of $\mathbb {R}^\mathcal {X}$. *Complexes* are the elements in the vector space $\mathbb {R}^\mathcal {X}$ of the form $y_R = s_1 e_{x_1} + \ldots + s_k e_{x_k}$ or $y'_R = s'_1 e_{x'_1} +\ldots + s'_{k'} e_{x'_{k'}}$, where the reaction

4.1 $$\begin{aligned} R:\qquad s_1 x_1 + \ldots + s_k x_k \rightarrow s'_1 x'_1 + \ldots +s'_{k'} x'_{k'} \end{aligned}$$

ranges over the reaction set $\mathcal R$. A reaction *R* as above defines an equivalence $y_R\sim y'_R$ in the set of complexes. Completing the set of equivalences $(y_R\sim y'_R,\ R\in \mathcal {R})$ yields a set of equivalence classes called *linkage classes*. Then the *deficiency index* of a network *G* is by definition

4.2 $$\begin{aligned} \delta = |{\text {Compl}}|-|{\text {Link}}|-s, \end{aligned}$$

where: ${\text {Compl}}$ is the set of complexes; ${\text {Link}}$ is the set of linkage classes; and *s* is the rank of the stoichiometric matrix.

### Definition 4.2

*(Weakly reversible)* If every pair of complexes in a linkage class can be strongly connected through some sequence of reactions $\in \mathcal {R}$, the network is called weakly reversible.

Note that all five types of diluted autocatalytic cores are both, weakly reversible and irreducible.

The Deficiency Zero Theorem proved by M. Feinberg (see Feinberg (1987), or Feinberg (1995), Theorem C.1 (ii)) states in particular that *non-degenerate weakly reversible networks with deficiency index *$\delta =0$*admit a unique positive stationary state *$C_{stat}$, and that this state is *asymptotically stable*. (For degenerate networks, one typically finds that the set of stationary solutions is a union of hyperplanes of positive dimension, whose points are indexed by stoichiometric compatibility classes, or elements in (rk$( {\mathbb S}))^{\perp }$, see Feinberg 1995, Definition 3.3). Note that the asymptotic stability is based on the existence of an explicit Lyapunov function for the dynamical system,

4.3 $$\begin{aligned} h(C) := \sum _{x\in \mathcal {X}} C_{stat,x} \Big ( \frac{C_x}{C_{stat,x}} (\ln (\frac{C_x}{C_{stat,x}})-1) + 1 \Big ), \end{aligned}$$

namely, *h* is strictly decreasing on integral curves. If all reactions are 1–1, then $\sum C_x(t)$ is a constant by probability conservation (which may be chosen equal to 1), *h*(*C*) is the relative entropy of the probability measure *C* with respect to the stationary probability measure $C_{stat}$. This is a well-known standard result in Markov theory, we expand upon it in Appendix section 7.3 (In the context of master equation systems, see Schnakenberg (1976), section V).

### Deficiency zero argument

Consider the reversible extension of a diluted autocatalytic core. It can be checked that, for all types, $s=|\mathcal {X}|$ (the stoichiometric matrix has full rank). We prove the following:

#### Lemma 4.3

The deficiency index of all diluted autocatalytic cores is zero.

#### Proof

The previous classification allows an easy computation of the deficiency index.

Type I: in case of a unique replication reaction $x_n\rightarrow s_n x_1$, one gets: ${\text {Compl}}=\{e_{x_1},\ldots ,e_{x_n},s_n e_{x_1}\}$, $|{\text {Compl}}|=n+1$, $s=n$, $|{\text {Link}}|=1$ (all complexes are in the same linkage class), so that $\delta =0$. If $x_k\rightarrow s_k x_{k+1}$ $(1\le k\le n-1)$ with $s_k\not =1$ is also a replication reaction, then the two linkage classes are 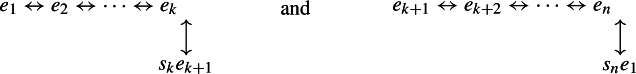 In general, any extra replication reaction increments the cardinal of Compl and the cardinal of Link, so this has no effect on the value of $\delta$.

Type II: We may assume that all stoichiometric coefficients on $\mathcal C$ are equal to 1 (as in the case of Type I, having replication reactions along $\mathcal C$ has no effect on $\delta$). Assume first that $\ell =1$, then ${\text {Compl}}= \{e_2,e_3,\ldots ,e_n,e_1, e_2+ e_{\sigma _1}\}$ and $|{\text {Link}}|=1$, so that $\delta =0$. If $\ell =2$, then ${\text {Compl}}$ also includes $e_{i_2+1}+e_{\sigma _2}$, and the two linkage classes are 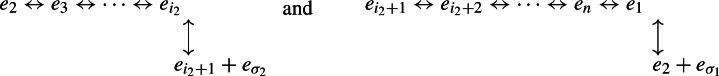


In general, any back branch increments the cardinal of Compl and the cardinal of Link, so this does not affect the value of $\delta$.

Type III: following the two cycles, one gets ${\text {Compl}}= \{ e_u,\ldots ,e_v\}\cup \{e_x,\ldots ,e_u\}\cup \{e_{x'},\ldots , e_u\} \cup \{e_x + e_{x'}\}$, so that $|{\text {Compl}}|=|\mathcal {X}|+1$. Since there is only one linkage class, $\delta =0$.

Type IV: $|{\text {Compl}}|=|\mathcal {X}|+2$ (due to the two one-to-many reactions), and correspondingly, two linkage classes 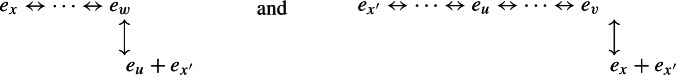 Type V: $|{\text {Compl}}|=|\mathcal {X}|+3$ (due to the three one-to-many reactions), and correspondingly, three linkage classes 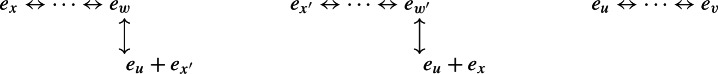
$$\square $$

#### Remark

*(Deficiency with degradation)* Consider a core $G=(\mathcal {X},\mathcal {R})$. We now assume that all species can be degradated, i.e. all degradation rates $a_x, x\in \mathcal {X}$ are $>0$. This changes totally the count in the deficiency index, since all linkage classes defined for zero degradation are now connected to the zero complex, whence |Link|=1, whereas the rank *s* of the stoichiometric matrix is left unchanged. Recall that Complexes are elements in the vector space $\mathbb {R}^\mathcal {X}$; the zero complex is the zero vector. Even though including it in the network changes the complexes and the linkage classes, it does not affect the rank of its reaction vectors (See Chapter 3 of Feinberg (2019)). Thus, if $|\mathcal {R}_{>1}|$ is the number of one-to-many reactions (note that all product composition $y_R, R\in \mathcal {R}_{>1}$ are distinct),

4.4 $$\begin{aligned} \delta = (|\mathcal {X}| + |\mathcal {R}_{>1}| + 1) - 1 - |\mathcal {X}| = |\mathcal {R}_{>1}|. \end{aligned}$$

Thus: $\delta \ge 1$ always; the extended Deficiency One Theorem (see Theorem A.1 in Feinberg (1995)), implying (for cores) uniqueness (but not existence) of stationary states, applies if and only if $\delta =1$; and finally, $\delta =1$ only in the following cases:

Type I (minimal); $\hbox {Type II}_{\ell =1}$ (minimal); Type III

where minimal means: minimal stoichiometric coefficients (all equal to 0 or 1, except in the case of Type I, where a single replication reaction $B_n\rightarrow mB_1$, $m\ge 2$ is allowed). Thus a different approach is taken for this case (Section 5).

### Note on Persistence and the Global attractor conjecture

The *Persistence conjecture*(Feinberg 1989) states that trajectories of the mass action system originating in the positive orthant cannot get arbitrarily close to the boundary if the system is weakly reversible. For deficiency zero systems, this also implies the *Global Attractor Conjecture* (Horn 1974), which in our case adds to the Deficiency zero theorem the fact that the unique stationary state is globally attracting, i.e. the solution of the dynamical system $dC/dt = {\mathbb S} J(C)$ converges to $C_{stat}$ as $t\rightarrow \infty$ for any positive initial condition.

Reference (Deshpande and Gopalkrishnan 2014) introduced the notion of Siphons,

#### Definition 4.4

*(Siphon)* See (Deshpande and Gopalkrishnan 2014) Let $G=(\mathcal {X},\mathcal {R})$ be a reaction network. A set $T\subseteq \mathcal {X}$ of species is a siphon iff for every reaction $(y_R\rightarrow y'_R,\ R\in \mathcal {R})$, if the complex $y'$ contains a species from *T*, then the complex *y* contains a species from *T*

In the case of reversible extensions of dilute autocatalytic cores, the minimal siphon is $\mathcal {X}$ itself, and it is also self-replicable and drainable (there exists sequence of reactions that increase, decrease resp. the amount of all species). In such a case, persistence is the self-replicable nature "overcoming" the drainability, but these notions are still vague in the literature, and one of the key necessities towards the *Global attractor conjecture* from the perspective of siphons (see Remark 6.4 in Deshpande and Gopalkrishnan (2014)).

On the other hand, our case is simpler for the five core types. We cannot have a boundary steady state which is not all 0 (Lemma 5.2 in the following section), and near the zero stationary state, the only one on the boundary, the total concentration of the system can only increase (refer to Appendix 7.4 for explicit calculation of the Jacobian determinant). Hence, it is unstable and trajectories cannot converge to it, proving the *Global attractor conjecture* for reversible extensions of autocatalytic cores. The trajectories stay bounded in the positive space, and must converge to the global attractor. This consideration, along with Lemma 4.3 proves Theorem 4.1.

## Uniqueness of states with degradation rates

Consider a core $G=(\mathcal {X},\mathcal {R})$. We now assume that all species can be degradated, i.e. all degradation rates $a_x, x\in \mathcal {X}$ are $>0$. First, we prove two short, but important lemmas, 5.1 and 5.2, that form a part of the hypothesis for Theorem 5.1. Then we state the main result for the uniqueness of the stationary states of cores in the presence of degradation and demonstrate the proof. Then we demonstrate that the assumption of non-degenerate zero stationary state at vanishing degradation is valid for the cores. Finally the last sub-section gives an example of a multi-stationary network with two one-to-many reactions.

There already exists works investigating how degradation rates affect steady states, but they have to do with inheriting the multistationary nature of the subnetworks (Banaji and Pantea 2018; Cappelletti et al. 2019; Craciun and Feinberg 2006; Joshi and Shiu 2012). Our case differs in two ways. The inheritance of monostationarity requires proving that no new stationary states can arise for some set of reaction rates for the old and the added reactions, while for multistationarity a proof that at least two stationary states get ’inherited’ for some set of rates suffices. The second is that, unlike settings where inflows and outflows are added together (Craciun and Feinberg 2006 for example), we only add an outflow for each species. The topological form of autocatalytic cores makes it so that it does not, in general, fit the assumptions of existing theorems that address monostationarity either directly or as absence of multistationarity. Thus, a new investigation is required, as described in this Section.

### No non-zero stationary state for large degradation rates

Let $(\alpha _x)_{x\in \mathcal {X}}$ be the degradation rates. A direct reaction *R* is of the form $x\rightarrow \cdots$, therefore $y_R=e_x$; by abuse of notation, we write $k_{x\rightarrow y'}$ instead of $k_{e_x\rightarrow y'}$. Conversely, a reverse reaction *R* is of the form $\cdots \rightarrow x'$, therefore $y'_R=e_{x'}$, and we write then $k_{y\rightarrow x'}$ instead of $k_{y\rightarrow e_{x'}}$. One-one reactions *R* are such that $||y||_1:= \sum _x y_x = 1$ and $||y'||_1=1$; all other reactions have either $||y||_1>1$ or $||y'||_1>1$ (but not both). An expression like $\sum _{x\rightarrow y'} f(x,y')$ is to be understood as the sum of the values $f(x,y')$ for all reactions $R:e_x\rightarrow y'$ with *x* fixed.

#### Lemma 5.1

Let $K:=\max _{x\rightarrow y' \ |\ ||y'||_1>1} k_{x\rightarrow y'}$ and $M:= \max _x \sum _{x\rightarrow y'} (||y'||_1-1)$. Then, if

5.1 $$\begin{aligned} \forall x\in \mathcal {X}\, \qquad \alpha _x\ge KM \end{aligned}$$

there is no strictly positive stationary state.

#### Proof

Let $$C>0$$. Then

5.2 $$\begin{aligned} \langle 1, \frac{dC}{dt}\rangle = \sum _x [x] \Big ( \sum _{x\rightarrow y'} k_{x\rightarrow y'} \langle 1,y'-e_x\rangle - \alpha _x\Big ) + \sum _{x'} \sum _{y\rightarrow x'} k_{y\rightarrow x'} [y] \langle 1,e_{x'}-y\rangle . \end{aligned}$$

The second sum is $<0$ since $\langle 1,e_{x'}-y\rangle = -(||y'||_1-1)\le 0$, more precisely $<0$ for all one-to-many reactions. The factor between parentheses in the first term is $\sum _{x\rightarrow y'} k_{x\rightarrow y'} (||y'||-1) - \alpha _x \le KM-\alpha _x\le 0$. $\square$

### No stationary state on the boundary

The *positive* *orthant* is defined as the space $\mathbb {R}_+=\{x\,|\,[x_i]>0\,\forall \,x_i\in \mathcal {X}\}$

#### Lemma 5.2

For any of the 5 types of cores ($G=(\mathcal {X},\mathcal {R})$, with or without degradation), there cannot be a stationary state with some concentrations 0 but not all (i.e. on the "boundary" of the positive orthant)

#### Proof

Let the set of species with vanishing concentrations be $\mathcal {B}\subseteq \mathcal {X}$. Note that for any species $x_i\in \mathcal {X}$, the possible types of outgoing reactions for any of the cores (for some other species $x_j,x_k\in \mathcal {X}$) are

- $$x_i \rightarrow \emptyset /x_j/x_j +x_k$$
- $$x_i+x_j\rightarrow x_k$$
- $$2x_i\rightarrow x_j$$

No other type of reaction contributes to the decrease in the concentration of $x_i$, and hence consequently, in the mass action equation for $d[x_i]/dt$, all other terms are positive. The latter takes the general form

5.3 $$\begin{aligned} \frac{d[x_i]}{dt}=-[x_i](\sum _{X_j\in \mathcal {X}}s_j[x_j]+s)+f([x_j]_{X_j\ne X_i}) \end{aligned}$$

where some $s_j,s$ may be 0 but some are positive, and *f* is a polynomial of the rest of the concentrations with all coefficients positive.

Now, consider $x_i\in \mathcal {B}$, i.e. $[x_i]=0$ at the stationary state. We get from (5.3) that $f=0$, which in turn implies that for any species $x_j$, which is the reactant of a reaction with $x_i$ as a product (or one of), $[x_j]=0$ and thus $x_j\in \mathcal {B}$. Now, for each autocatalytic core *G*, we define a Directed Graph $G'=(\mathcal {X},E')$, where

- Edge $$\{x_i,x_j\},\{x_j,x_i\}\in E'$$ if $$x_i\rightleftarrows x_j\in \mathcal {R}$$
- Edge $$\{x_j,x_i\},\{x_k,x_i\}\in E'$$ if $$x_i\rightleftarrows x_j+x_k\in \mathcal {R}$$

Note that $\{x_i,x_j\}\in E'$ and $x_i\in \mathcal B$ $\implies x_j\in \mathcal B$. Since $G'$ defined for each core is a weakly connected digraph, we get $\mathcal {B}=\mathcal {X}$, the zero stationary state. $\square$

### General uniqueness of stationary states

#### Theorem 5.1

Take a chemical reaction network of *n* species, $G=(\mathcal {X},\mathcal {R})$ and consider $G'=(\mathcal {X},\mathcal {R'})$ to be the extension of G with added non-zero degradation reactions for each species $x\in \mathcal {X}$. Let [*x*] denote the concentration of species *x* and *C* is the vector of concentrations. Let the network satisfy mass action kinetics with the following assumptions:

1. *G* is a weakly reversible deficiency 0 network
2. Neither *G* nor $G'$ has any stationary state on the boundary of the positive orthant except the zero state
3. There exists a continuous function $m_b(a,k)$, such that any positive stationary state of $G'$ with kinetic rates *k* and degradation rates *a* satisfies $m_b(a,k)\ge \max \{[x],x\in \mathcal {X}\}$
4. There are no degenerate non-zero stationary states for $G'$ for all choices of parameters
5. The Jacobian (defined below) of the mass action equations of $G'$ at the zero state is 0 for exactly one value of $\alpha \ge 0$, if we take degradation rates of the form $\alpha .a_i$,$\alpha \in \mathbb {R}^+$ for each species $x_i\in \mathcal {X}$, fixing $a_i\in \mathbb {R}^+$

Then, the network $G'$ has at most one non-zero stationary state for any values of kinetic and degradation rates.

#### Proof

By Assumption 1 and deficiency theory, *G* has only one non-zero stationary state, which is asymptotically stable. Assume that there exists a set of rates *k* and degradation values *a*, for which $G'$ has at least two positive stationary states. We consider the degradation rates in a variable form: $(\alpha .a)$,$\alpha \in \mathbb {R}^+$ and prove contradiction with the hypotheses by letting $\alpha$ vary. $\square$

Let $f(\alpha .a,C)$ denote the mass action equations of the system for these fixed kinetics.

Let $S(\alpha )$ denote the set of solutions in *C* of the mass action equations $f(\alpha .a,C)$ with $C\ge 0$. Let $S^*(\alpha )=S(\alpha )\cap \{C>0\}$.The Jacobian matrix

$$ {\textbf {J}}(\alpha .a,C)=\left[ \begin{array}{cccc} \frac{\partial {\textbf {f}}}{\partial x_1}&\frac{\partial {\textbf {f}}}{\partial x_2}&\dots&\frac{\partial {\textbf {f}}}{\partial x_n} \end{array} \right] $$

is the (square in this case) matrix of all the first-order partial derivatives of the equations, and its determinant is referred to as the Jacobian Determinant. The matrix is invertible if the determinant is non-zero. A state *C* is called degenerate if $C\in S(\alpha )$ and det $J(\alpha .a,C)=0$.

Take *M* as the bound defined by Lemma 5.1. At $\alpha =1$ there are at least two non-zero stationary states, and at $\alpha =M$ there are none. Also by Assumption 5, there are no degenerate stationary states apart possibly from the origin for $0<\alpha \le M$.

Considering the bound on the solutions based on assumption 3, the solutions for any $\alpha \le M$ are bounded by $\max \{m_b(\alpha .a',k)$ | $0\le \alpha \le M\}$ and cannot diverge. Call this compact subset $\mathcal {V} \subset \mathbb {R}^{n}$. Let $\mathcal {V}^+=\mathcal {V}\cap \mathbb {R}^{n+}$

We start with a short Lemma,

#### Lemma 5.3

(Maximal solution of C) Let $C_{ss}\in S^*(\alpha ^o)$ be non-degenerate,

Define $I_{max}=\uplus \{I$ | $\alpha ^o\in I, \exists$ $C^o_{st} (\alpha ):I\rightarrow \mathcal {V}$ satisfying conditions (*),(**),(***)},

(*) $$C^o_{st}(\alpha ^o)=C_{ss}$$

(**) $$\forall \alpha \in I$$, $$C^o_{st}(\alpha )\in S^*(\alpha )$$

(***) $$\forall \alpha \in I$$, det $$J(\alpha ,C^o_{st}(\alpha ))\ne 0$$,

Then there exists a unique $C_{st}(\alpha ):I_{max}\rightarrow \mathcal {V}$ such that it satisfies (*),(**),(***) for $I_{max}$

#### Proof

If $C_{ss}$ is a stationary state of the system $G'$ at $\alpha ^o$ with an invertible Jacobian matrix, then, by the implicit function theorem, there exists an open interval $I^o$ around $\alpha ^o$ and a unique continuous function $C_{st}:I^o\rightarrow \mathcal {V}^+$ such that $C_{st}(\alpha ^o)=C_{ss}$ and $C_{st}(\alpha )$ $\in S(\alpha )$ for all $\alpha \in I^o$. Thus $I_{max}$ is non-empty.

If $I^a$ and $I^b$ are two intervals with continuous functions $C_{st}^a(\alpha )$ and $C_{st}^b(\alpha )$ on the intervals (respectively) satisfying (*),(**),(***), with $C_{st}^a(\alpha ^o)=C_{st}^b(\alpha ^o)=C_{ss}$,

Let $I'=\{\alpha$ | $C_{st}^a(\alpha )=C_{st}^b(\alpha )\}$, then $I'$ is open in $I^a\cap I^b$ because of (***), and it is closed by continuity; thus (by connectedness) $I'=I^a\cap I^b$.

Setting $C_{st}(\alpha )=$ $${\left\{ \begin{array}{ll} C_{st}^a(\alpha ) \text { if } \alpha \in I^a \\ C_{st}^b(\alpha ) \text { if } \alpha \in I^b \end{array}\right. }$$, we get a function continuous on the set $I^a\cup I^b$

Gluing all such intervals together gives a unique continuous function $C_{st}$ over $I_{max}$. $\square$

As there are at least two distinct stationary states at $\alpha =1$, select any two distinct states $C_{ss}^1$ and $C_{ss}^2$, and define as in the Lemma 5.3, the intervals $I_{max}^1$ and $I_{max}^2$ and continuous functions $C^1_{st}:I^1_{max}\rightarrow \mathcal {V}$ and $C^2_{st}:I^2_{max}\rightarrow \mathcal {V}$, such that $C^i_{st}(1)=C^i_{ss}$ for $i=1,2$. We exhibit a contradiction by considering the behaviour of functions $C^i_{st},i=1,2$ at the end points of $I'=I_{max}^1\cap I_{max}^2=(\alpha _{min},\alpha _{max})$.

Let $\alpha '=\alpha _{min}$ or $\alpha _{max}$; by compactness there exists a sequence $\alpha _n$ in $I'$ converging to $\alpha '$, such that the sequence ($C_{st}^1(\alpha _n)$,$C_{st}^2(\alpha _n)$) converge in $\mathcal {V}^2$. Let ($C_{st}^1(\alpha ')$,$C_{st}^2(\alpha ')$)=$\lim _{n\rightarrow \infty }(C_{st}^1(\alpha _n),C_{st}^2(\alpha _n))$.

Consider first $\alpha '=\alpha _{max}$; if $C_{st}^1(\alpha _{max})\ne C_{st}^2(\alpha _{max})\ne 0$, by Assumption 4 we can apply the implicit function theorem again to extend the interval $I'$, contradicting the maximality of $\alpha _{max}$. Thus either $C_{st}^1(\alpha _{max})=0$ and det $J(\alpha _{max},C_{st}^1(\alpha _{max}))=0$, or $C_{st}^2(\alpha _{max})=0$ and det $J(\alpha _{max},C_{st}^2(\alpha _{max}))=0$. This implies that det $J(\alpha _{max},0)=0$

Now consider $\alpha '=\alpha _{min}$. If $\alpha _{min}>0$, we get again det $J(\alpha _{min},0)=0$, which is contradictory with Assumption 5 as $\alpha _{min}\ne \alpha _{max}$; thus $\alpha _{min}=0$. Also note that $C_{st}^1(\epsilon ),C_{st}^2(\epsilon )\ne 0$ for $0<\epsilon <\alpha _{max}$

The origin is non-degenerate (by Assumption 5 again) and the bulk stationary state (call it $C_{\delta }$) is also non-degenerate (Theorem 15.2.2 from Feinberg (2019)).

If $C_{st}^{1,2}(\alpha _{min})$=$C_{st}^{1,2}(0)=C_{\delta }$, it violates the uniqueness of the continuous function we would get by applying the implicit function theorem at $C_{\delta }$. If $C_{st}^{1/2}(0)=0$, along with the constant function $C(\alpha )=0$, it violates the uniqueness of the implicit function at $C=0$.

Thus the existence of the two stationary states $C^1_{ss}\not =C^2_{ss}$ at $\alpha =1$ is contradictory with the hypotheses of the Theorem.

#### The zero stationary state for vanishing degradation rates

The Jacobian of a core at vanishing degradation and zero stationary state (*J*(0.*a*, 0)) is identical to the Jacobian of the dynamical core as classified in Section 3, but with all one-one reactions made reversible. Denote the latter chemical reaction network by $G=(\mathcal {X},\mathcal {R})$.

We use the child selection formalism developed in Vassena (2020) to determine the properties of the Jacobian of this system.

For any reaction $r\in \mathcal {R}$, let $\bar{r}$ denote the reverse reaction, with reactant and product species interchanged.

The reaction network of *n* species, *G*, has a form where for all species $x\in \mathcal {X}$ there exists either 1 or 2 outgoing reactions with a single reactant (which is *x*). *t* reactions in $\mathcal {R}$ are one-to-many reactions, and all the other reactions are in the form of $(n-t)$ reversible pairs of 1-1 reactions, *r* and $\bar{r}$ (total $2n-2t+t$ reactions). Furthermore, there exists a species $x\in \mathcal {X}$, with only one outgoing reaction, which is also a one-one reaction.

A child selection is an injective map **Cs: **$\mathcal {X}\rightarrow \mathcal {R}$, which associates to every species $x\in \mathcal {X}$ a reaction $r\in \mathcal {R}$ such that *x* is a reactant species of reaction *r*. Then for the Jacobian matrix *J*,

5.4 $$\begin{aligned} det\,J=\sum _{Cs} det\,S^{Cs} \prod _{x\in \mathcal {X}} \frac{\partial k_{Cs(x)}}{\partial x} \end{aligned}$$

$S^{Cs}$ is the $n\times n$ matrix whose $i^{th}$ column is the $Cs(i)^{th}$ column of the stoichiometric matrix *S*, $k_{Cs(x)}$ is the mass action rate of reaction *Cs*(*x*), and the sum runs over all possible child selections *Cs*.

In our particular case, any child selection maps the *n* species to *n* reactions out of $2n-t$. If $Cs(\mathcal {X})=\{Cs(x): x\in \mathcal {X}\}$ contains both *r* and $\bar{r}$, then *det* $S^{Cs}=0$.

A child selection with a non-zero contribution to *det* *J* must have all *t* one-to-many reactions and $(n-t)$ 1-1 reactions while avoiding reversible pairs.

When all the one-to-many reactions are included, there is only one assignment of the 1-1 reactions possible, and it is exactly the form of the reactions for the core in Section 3. The contribution for this child selection is non-zero, as $S^{Cs}$ is irreversible (by a case-by-case check) by the hypothesis of Section 3, and all partial derivatives of the mass action rates for these reactions are positive constants. Thus the Jacobian determinant is non-zero. This is shown explicitly for each type in Appendix 7.4.

It is easy to see that the only possibility assigns to each *x* the reaction outgoing from *x* as depicted in the irreversible graphs of section 3.

##### Theorem 5.2

All autocatalytic cores (apart from Type $II_l$ with $\ell >2$), with strictly non-zero degradation, can have at most one positive stationary state

##### Proof

Type I and Type III can be explicitly solved to get the uniqueness of stationary states, as demonstrated in Supplementary Information Section S3

For the rest of the cores, they satisfy all the conditions of Theorem 5.1, and hence can have at most one non-zero stationary state. Section 4 gives condition 1, 5.2 gives condition 2, 5.3 gives condition 5, and conditions 3,4 are explicitly demonstrated for each case in the Supplementary Information section S4. Type $II_l$ with $l>2$ are not included in the proof and the uniqueness of their stationary state with degradation remains an open question. $\square$

##### Remark

*Some degradations zero, some non-zero* In the case of some degradation rates vanishing but not all, our proof of uniqueness still holds for all types except Type V and $\hbox {Type II}_{l>2}$ (See Section S3 and Section S4.3 in Supplementary information).

### Multistationarity for interaction of multiple cores

We end this section with a example of how we can have multistationarity. This example is a slightly modified version of Example 2.3 in Joshi et al. (2023). Species $X_3$ and $X_4$ for a Type I core, and it has been coupled to a fork with $X_1$ and $X_2$. The inputs to $X_1$ and $X_2$ can again be viewed as a coupling to an out reaction (degradation) of an autocatalytic core at equilibrium.

5.5 $$\begin{aligned} X_1 \rightleftarrows X_2 +X_3 \nonumber \\ X_3 \overset{16}{\rightleftarrows }\ X_4 \nonumber \\ 2X_3 \overset{2}{\rightleftarrows }\ X_4 \nonumber \\ 0 \overset{6}{\rightleftarrows }\ X_1 \nonumber \\ 0 \overset{27}{\rightleftarrows }\ X_2 \end{aligned}$$

Fix the unspecified rates as 1, this system can be solved to show that it admits three distinct positive stationary concentrations

$$[x_1]=18,[x_2]=15,[x_3]=2,[x_4]=20$$

$$[x_1]=13,[x_2]=20,[x_3]=1,[x_4]=9$$

$$[x_1]=21,[x_2]=12,[x_3]=3,[x_4]=33$$

The first solution is stable while the latter two are unstable.

Example 2.6 from Joshi et al. (2023) is also a multistationary system, which involves a non-linear coupling reaction between two autocatalytic reactions.

## Discussion

In this work, we characterized minimal autocatalytic networks, called autocatalytic cores. Our two main results are that: (i) Stoichiometric autocatalytic cores are identical to dynamical autocatalytic cores in the diluted regime. (ii) Dynamical autocatalytic cores have a single stationary state.

A reaction is stoichiometrically autocatalytic if there exists a set of reaction rates such that every species of the network is positively produced (Blokhuis et al. 2020), provided that every reaction has at least one reactant and one product, a condition called *autonomy*. Equivalently, the image of the stoichiometric matrix, which has plus and minus signs in every column, intersects the strictly positive orthant. Minimal stoichiometries allowing autocatalysis, or stoichiometric autocatalytic cores, were classified into five types (Blokhuis et al. 2020), which differ by the number of one-to-many reactions they possess and the entanglement of their internal catalytic cycles. In this regard, the Type II cores are special as they are the only type where the number of catalytic cycles *i* varies, leading to subcategories denoted $II_i$. For all types, each category or sub-category comprises an infinity of variants generated by the addition of intermediate reaction steps having a single species on each side with the same multiplicity. By definition of minimality, any autocatalytic system must contain an autocatalytic core. Thus, possessing an autocatalytic core is a necessary condition for autocatalysis, which has been used to detect autocatalysis in real chemistries (Peng et al. 2022). We should also mention that more relaxed and more restricted stoichiometric criteria have been proposed in the literature (Hordijk et al. 2017; Andersen et al. 2021; Deshpande and Gopalkrishnan 2014), with Type II corresponding to minimal autocatalytic sets as defined in Steel et al. (2013).

The present work deals with dynamical autocatalysis, following up on Unterberger and Nghe (2022), as autocatalysis is ultimately a dynamical property (Lin et al. 2020; Horváth 2021). Stoichiometric autocatalysis does a priori not guarantee dynamical autocatalysis, because the required reaction vectors may not be compatible with kinetic laws (Lin et al. 2020), or degradation rates are too high as compared to the production rate of species, thus turning the zero zero-concentration state into an attractor. The kinetic viability of autocatalytic networks has been studied on a case-by-case basis (Blokhuis et al. 2020), but there is currently no general result characterizing dynamical autocatalysis for arbitrary degradation rates of each species. However, in the limit regime of diluted reaction networks, reference (Unterberger and Nghe 2022) shows that there exist finite values of degradation rates, for which networks with the topological property (Top) possess a positive Lyapunov exponent, meaning a positive exponential growth rate. Colloquially, (Top) is equivalent to requiring that the strongly connected components, which have no inflows from other components, possess at least one one-to-many reaction. One may then consider minimal networks allowing dynamical autocatalysis in the diluted regimes, here called *dynamical autocatalytic cores*, which corresponds to minimal networks verifying (Top). We showed here that dynamical cores follow the same classification as that of stoichiometric cores. Specifically, reversible extensions of dynamical cores belong to the same five types as stoichiometric cores, but are the subset where species can have a multiplicity larger than 2 only if they are products along Type I cores. This guarantees that autocatalytic systems capable of exponential growth starting from zero or small concentrations (given small degradation rates) are exactly those detected using stoichiometric cores.

The eigenvector associated with the Lyapunov exponent, up to normalization, gives the fraction of each species during exponential growth, called the *chemical composition*. In practice, this regime may be realized at intermediate times in large closed chemical reactors, where the growth mode associated with the Lyapunov exponent becomes dominant compared to all other modes, but the accumulation of products does not yet cause visible non-linearities. This type of behaviour is well-known in microbiology, where growth in the bioreactor is typically characterized by three phases leading to a sigmoidal profile (called ’Monod’ growth): an initial phase that appears as a delay (’lag’ phase), a stationary exponential growth phase (’log’ phase), and a saturation (’stationary’ phase). The growth mode associated with the Lyapunov exponent can also be observed in open reactors, according to several modalities. One is the so-called CSTR, for a continuously stirred chemical reactor, where reactants consumed by the reaction are injected at a constant flux, the reactor is actively mixed, and some of the solution is extracted at an equal rate in order to maintain the total volume constant. CSTRs can be approximated by serial dilution protocols, where a finite fraction is extracted from a reactor after a given incubation time, and then injected in fresh solution at regular time intervals. The composition approaches the Lyapunov eigenvector one as the incubation time gets smaller (Blokhuis et al. 2018). Yet another modality is the chemostated open reactor, meaning that the concentration of reactants is maintained fixed, and the dilution rate is matched with the growth rate. Although theoretically convenient, this type of reactor requires semi-permeable boundaries. This situation is in fact realized in living cells, as these are equipped with elaborated import pumps and osmotic regulation mechanisms that maintain the total concentration constant. However, it was shown recently that this type of mechanism could be realized in the absence of evolved bio-molecules, by transport phenomena and osmosis in a rudimentary multi-phase system containing autocatalytic reactions (Lu et al. 2023).

As products accumulate, many-to-one and many-to-many reactions start to participate in the dynamics. Reverse reactions should then be considered, resulting in non-linear kinetics, making it non-obvious whether the Lyapunov mode is the only stationary growth regime. Multistationarity is of particular interest for emergent properties in reaction networks (Novichkov et al. 2021), notably in the context of the origin of life, where multiple growth modes in competition would enable rudimentary forms of Darwinian evolution (Ameta et al. 2022; Adamski et al. 2020; Pavlinova et al. 2022). Here, multistationarity is understood in the composition space (the fraction of each species), which in a chemostated system corresponds to actual multistationarity in concentration, as well as in homeostatic systems that grow while maintaining a constant total concentration (as cells during exponential growth) (Sughiyama et al. 2022). Formally, the reverse reactions lead us to consider what we termed the *reversible extension* of the network. In the absence of degradation, deficiency theory provides a direct argument showing the uniqueness of a stationary composition (Feinberg 2019). However, this theory is not directly applicable when adding arbitrary degradation rates. By using direct computations of stationary regimes and interpolating the behaviour between large degradation rates and zero degradation, we could show the uniqueness of the stationary regime for almost all possible rate constants of the mass action kinetics. This result was obtained for Type I, $\hbox {Type II}_{1,2}$, Type III, Type IV and Type V. We could not establish the same result for $\hbox {Types II}_>2$; however numerical tests suggest that the same result applies. Thus, it remains an open question to address the uniqueness of the stationary state for these types, which correspond to minimal networks in the theory of collective autocatalytic sets.

Autocatalytic motifs were shown to be among the possible stoichiometries prone to trigger instabilities (Vassena and Stadler 2024), which in our context means that the zero concentration state is an unstable stable solution, but the bulk solution (where all species of the autocatalytic core have positive concentrations) is the only possible stable one. The fact that a stationary state is unique implies that phenomena richer than growth must proceed through an interplay between different Types of minimal cores and other types of negative/positive feedbacks, or more complicated kinetics that cannot be simplified into mass-action type equations. This uniqueness also means that autocatalytic cores alone lead to growth that is robust to perturbations, which is a potential advantage for primordial metabolism (Ameta et al. 2021).

## Supplementary Information

Below is the link to the electronic supplementary material.

Supplementary file 1 (pdf 341 KB)

## Data Availability

Data sharing not applicable to this article as no datasets were generated or analysed during the current study.

## Appendix

The first subsection gives practical examples of the cores we get in the classification from metabolic networks. Section 7.2 contains two definitions used in Section 3. Section 7.3 expands upon the Lyapunov function for diluted autocatalytic cores, and Section 7.4 is an explicit calculation for the Jacobian Determinant of the five cores with degradation at the zero steady state, showing that it is always non-zero.

### Examples of autocatalytic cores from metabolism

#### Example 1

*Reverse Krebs cycle* (see e.g. Smith and Morowitz (2016), Fig. 4.1). It is presented as Type II in Blokhuis et al. (2020); it may actually be seen as a degenerate case of Type III (see above) with $x=u$. The path from *u* to *v* is the main anabolic path leading from Oxaloacetate (4 carbon atoms) to Citrate (6), through Succinate and OxaloSuccinate, by a succession of enzymatic reactions (reducative carboxylations, hydratations/dehydratations, reductions). The fragmentation reaction $v\rightarrow x+x'$ is the retro-aldolization Citrate $\longrightarrow$ Oxaloacetate + Acetate. Then the path from $x'$ to *u* is the anabolic path from Acetate (2) to Oxalocetate, which is homologous to the subpath of the main anabolic path leading from Succinate to OxaloSuccinate.

#### Example 2

*Glyoxylate cycle*, see. e.g. Moran et al. (2011), Fig. 13.27. The glyoxylate cycle, a less-known metabolic cycle used by plants, bacteria, protists and fungi to convert Acetyl-CoA to Succinate for glucid biosynthesis, is of Type III if Acetate is chemostatted. The path from *u* to *v* is the main anabolic path leading (through OxaloAcetate) from Malate (4 carbon atomes) to IsoCitrate (6), by a succession of enzymatic reactions (oxydations, aldolizations). The fragmentation reaction $v\rightarrow x+x'$ is the retro-aldolization IsoCitrate $\longrightarrow$ Glyoxylate + Succinate. Then, the addition of Acetate by aldolization leads from Glyoxylate (2) back to Malate, and (on the other branch), an oxydation of Succinate (4) to Fumarate (4), followed by hydratation, leads also back to Malate. Note that the second branch is not degenerate as in Example 1, but is not an anabolic path either (the number of carbon atoms is preserved).

#### Other Metabolic pathways

- *Type III*
  - an anabolic path from a shorter molecule *u* to a longer one *v* along $\mathcal {C}\cap \mathcal {C}'$;
  - followed by a fragmentation (e.g. retroaldolization) from *v* into short molecules *x* and $x'$;
  - and two anabolic paths, one from *x* along $\mathcal {C}$, one from $x'$ along $\mathcal {C}'$, leading both back to *u*.
- *Type IV*
  - a path of anabolic reactions and skeleton rearrangements leading from a short molecule $x'$ to *u* along $\mathcal {C}'$ , and then from *u* to a long molecule *v* (possibly, $v=u$) along $\mathcal {C}\cap \mathcal {C}'$;
  - a fragmentation (retroaldolization, etc.) of *v* into a shorter molecule *x* and directly back into $x'$;
  - and a path of anabolic reactions leading along $\mathcal {C}$ from *x* to a long molecule *w* (possibly, $x=w$), which is then fragmented back into *u* and into $x'$.
  Pictorially, with molecule sizes increasing upwards,  Paths from *u* to *v* and from *x* to *w* are depicted by a dashed line because they may have length 0 (case $u=v$ or $x=w$). All other paths (plain lines) have length $\ge 1$.

### Further Definitions

#### Definition 7.1

*(cycle)* Let $G=(\mathcal {X},\mathcal {R})$ be a network, $n\ge 2$, and $x_1,\ldots ,x_n\in \mathcal {X}$. Then $\mathcal {C}=\{x_1\rightarrow x_2\rightarrow \cdots \rightarrow x_n\rightarrow x_1\}$ is a cycle iff there exist split reactions $x_1\rightarrow x_2, x_2\rightarrow x_3,\ldots ,x_{n-1}\rightarrow x_n, x_n\rightarrow x_1$ going along the cycle. See Figure below. By abuse of notation, we write $x_{n+1}=x_1$.

A *split reaction along the cycle* is any split reaction $x_i\rightarrow x_{i+1}$ coming from a reaction in $\mathcal R$.

We write $i_1\prec i_2\prec \ldots \prec i_k$ if $x_{i_1}\not =x_{i_2}\not = \ldots \not =x_{i_{k-1}}$ and $x_{i_2}\not =x_{i_3}\not =\ldots \not =x_{i_k}$ (the case $i_1=1, i_k=n+1$, with the identification $x_{n+1}\equiv x_1$, is not excluded) and, starting from $i_1$ and going round the cycle once, one successively encounters $i_2,\ldots ,i_k$. (If $i_1=1$ this simply means $i_1=1<i_2<\ldots < i_k\le n+1$). Because the circle closes back onto itself, this is not an order relation (e.g. $1\prec n$ and $n\prec 1$). If, for some $j=1,\ldots ,k-1$, $|i_{j+1}-i_j|\not =1,n-1$ (i.e. if $i_{j+1},i_j$ are not neighbors), we write more precisely: $i_j \prec \prec i_{j+1}$.

#### Definition 7.2

*(ear)* Let $G=(\mathcal {X},\mathcal {R})$ be a network, $n\ge 2$, and $x_1,\ldots ,x_n\in \mathcal {X}$. Let $\mathcal {C},\mathcal {C'}$ be two cycles of *G*, such that $\mathcal {C}\ne \mathcal {C}'$ and $(\mathcal {C}\cap \mathcal {C}')\ne \phi$. Then an ear $\mathcal {O}$ is defined as $\mathcal {O}= \mathcal {C}\setminus (\mathcal {C}\cap \mathcal {C}')$.

### ’Hand-made argument’: Lyapunov function

Theorem C.1 (ii) in Feinberg (1995) is based on a general convexity inequality (Proposition 5.3), and on the fact that a certain relation holds in an abstract vector space $\mathbb {R}^\mathcal {C}$ with standard basis $(\omega _y)_{y\in {\text {Compl}}}$ indexed by the set of complexes, namely,

7.3.1 $$\begin{aligned} \sum _R \phi _{y_R\rightarrow y'_R}(C_{stat}) (\omega _{y'_R} - \omega _{y_R})=0. \end{aligned}$$

In general, only the stationary property, $\sum _R \phi _{y_R\rightarrow y'_R}(C_{stat})(y'_R-y_R)=0$ holds, which implies (7.3.1) by applying the linear operator

7.3.2 $$\begin{aligned} Y: \mathbb {R}^{{\text {Compl}}} \rightarrow \mathbb {R}^\mathcal {X}, \ \ \omega _y \mapsto y; \end{aligned}$$

precisely, it is proved in Feinberg (1995) that the kernel of *Y* restricted to span$(\omega _{y'_R}-\omega _{y_R}, R\in \mathcal {R})$ has dimension $\delta$; thus (7.3.1) follows if the deficiency index $\delta$ is zero. However, (7.3.1) is actually obvious if $C_{stat}$ is an equilibrium state; namely, letting $(R,\bar{R})$ be any couple (direct reaction, reverse reaction), the equilibrium property for $C_{stat}$ implies $\phi _{y_R\rightarrow y'_R}(C_{stat}) (\omega _{y'_R} - \omega _{y_R}) +\phi _{y_{\bar{R}}\rightarrow y'_{\bar{R}}}(C_{stat}) (\omega _{y'_{\bar{R}}} - \omega _{y_{\bar{R}}}) = (\phi _{y_R\rightarrow y'_R}(C_{stat}) -\phi _{y'_R\rightarrow y_R}(C_{stat}))(\omega _{y'_R} - \omega _{y_R}) =0$ by (2.11). Let us prove briefly that *h* is a Lyapunov function in this particular case for the sake of the reader. First it is well-known (and follows from strict concavity of the logarithm) that $h(C)\ge 0$ and vanishes only for $C=C_{stat}$, see e.g. Appendix C in Feinberg (1995). We let $\langle \, \cdot \, , \cdot \, \rangle$ be the standard scalar product in $\mathbb {R}^{\mathcal {X}}$. The generalized relative entropy function *h* satisfies the following identity,

7.3.3 $$\begin{aligned} \phi _{y\rightarrow y'}(C)= \phi _{y\rightarrow y'}(C_{stat}) \, \times \, \prod _{i=1}^n (C_i/C_{stat,i})^{y_i} = \phi _{y\rightarrow y'}(C_{stat}) \, \times \, e^{\langle y,\nabla h (C)\rangle } \end{aligned}$$

Now the convexity inequality $e^a (b-a)\le e^b - e^a,\ a,b\in \mathbb {R}$ implies

7.3.4 $$\begin{aligned} \frac{dh}{dt}= &  \langle \frac{dC}{dt}, \nabla h(C)\rangle \end{aligned}$$

7.3.5 $$\begin{aligned}= &  \sum _{y\rightarrow y'} \phi _{y\rightarrow y'}(C) \langle y'-y, \nabla h(C) \rangle \nonumber \\= &  \sum _{y\rightarrow y'} \phi _{y\rightarrow y'}(C_{stat}) e^{\langle y,\nabla h(C)\rangle } \, \langle y'-y, \nabla h(C) \rangle \nonumber \\\le &  \sum _{y\rightarrow y'} \phi _{y\rightarrow y'}(C_{stat}) \, (e^{\langle y',\nabla h(C)\rangle } - e^{\langle y,\nabla h(C)\rangle }) \nonumber \\= &  {1\over 2}\sum _{y\rightarrow y'} (\phi _{y\rightarrow y'}(C_{stat})- \phi _{y'\rightarrow y}(C_{stat})) \, (e^{\langle y',\nabla h(C)\rangle } - e^{\langle y,\nabla h(C)\rangle }) = 0. \end{aligned}$$

The second line comes from (2.12). The last line comes by antisymmetrizing and using (2.11), together with the fact that $C_{stat}$ is an equilibrium state. The inequality is strict except if $C=C_{stat}$.

### Non-degeneracy of the zero stationary state for vanishing degradation rates

Here we calculate the explicit Jacobian determinant at the zero stationary state of each core for no degradation, and show that it cannot be zero. Also assume that $C_{\delta }$ is a vector of very small non-negative concentrations, $([\delta x_i])_{x_i\in \mathcal {X}}$. The system near zero behaves as $\frac{dC}{dt}=J\times C_{\delta }$.

### Type I

The reactions are:

7.4.1 $$\begin{aligned} X_1 \overset{k_1^+}{\underset{k_1^-}{\rightleftarrows }} 2X_2 \qquad X_2 \overset{k_2^+}{\underset{k_2^-}{\rightleftarrows }} X_1 \end{aligned}$$

$$ J=\left[ \begin{array}{cc} -(k_1^++k_2^-) & k_2^+ \\ 2k_1^++k_2^- & -(k_2^+) \end{array} \right] $$

We get Det $J=-k_1^+k_2^+$, which is non-zero

At $C_{\delta }$, $\left. \left( \sum _{X_i\in \mathcal {X}}\frac{d[x_i]}{dt}\right) \right| _{C_{\delta }}=k_1^+[\delta x_1]\ge 0$. This inequality is also true for all of the following cases of types.

#### Type III

The reactions are:

7.4.2 $$\begin{aligned} X_1 \overset{k_1^+}{\underset{k_1^-}{\rightleftarrows }} X_2+X_3 \qquad X_2 \overset{k_2^+}{\underset{k_2^-}{\rightleftarrows }} X_1 \qquad X_3 \overset{k_3^+}{\underset{k_3^-}{\rightleftarrows }} X_1 \end{aligned}$$

$$ J=\left[ \begin{array}{ccc} -(k_1^++k_2^-+k_3^-) & k_2^+ & k_3^+ \\ k_2^-+k_1^+ & -k_2^+ & 0 \\ k_3^-+k_1^+ & 0 & -k_3^+ \end{array} \right] $$

And we get Det $J=k_1^+k_2^+k_3^+$, non-zero

#### Type II $l=1,2$

For one fork,

7.4.3 $$\begin{aligned} X_1 \overset{k_1^+}{\underset{k_1^-}{\rightleftarrows }} X_2+X_3 \qquad X_2 \overset{k_2^+}{\underset{k_2^-}{\rightleftarrows }} X_3 \qquad X_3 \overset{k_3^+}{\underset{k_3^-}{\rightleftarrows }} X_1 \end{aligned}$$

$$ J=\left[ \begin{array}{ccc} -(k_1^++k_3^-) & 0 & k_3^+ \\ k_1^+ & -k_2^+ & k_2^- \\ k_3^-+k_1^+ & k_2^+ & -(k_3^++k_2^-) \end{array} \right] $$

Again, we get a non-zero Det $J=k_1^+k_2^+k_3^+$

For two forks

7.4.4 $$\begin{aligned} X_1 \overset{k_1^+}{\underset{k_1^-}{\rightleftarrows }} X_2+X_3 \qquad X_2 \overset{k_2^+}{\underset{k_2^-}{\rightleftarrows }} X_6 \qquad X_3 \overset{k_3^+}{\underset{k_3^-}{\rightleftarrows }} X_1 \\ \nonumber X_4 \overset{k_4^+}{\underset{k_4^-}{\rightleftarrows }} X_5+X_6 \qquad X_5 \overset{k_5^+}{\underset{k_5^-}{\rightleftarrows }} X_3 \qquad X_6 \overset{k_6^+}{\underset{k_6^-}{\rightleftarrows }} X_4 \end{aligned}$$

$$ J=\left[ \begin{array}{cccccc} -(k_1^++k_3^-) & 0 & k_3^+ & 0 & 0 & 0 \\ k_1^+ & -k_2^+ & 0 & 0 & 0 & k_2^- \\ k_3^-+k_1^+ & 0 & -(k_3^++k_5^-) & 0 & k_5^+ & 0 \\ 0 & 0 & 0 & -(k_4^++k_6^-) & 0 & k_6^+ \\ 0 & 0 & k_5^- & k_4^+ & -(k_5^+) & 0 \\ 0 & k_2^+ & 0 & k_4^++k_6^- & 0 & -(k_6^++k_2^-) \end{array} \right] $$

Which yields Det $J=-k_1^+k_2^+k_3^+k_4^+k_5^+k_6^+$, always non-zero

#### Type IV

7.4.5 $$\begin{aligned} X_1 \overset{k_1^+}{\underset{k_1^-}{\rightleftarrows }} X_2+X_3 \qquad X_2 \overset{k_2^+}{\underset{k_2^-}{\rightleftarrows }} X_5 \qquad X_3 \overset{k_3^+}{\underset{k_3^-}{\rightleftarrows }} X_4 \\ \nonumber X_5 \overset{k_5^+}{\underset{k_5^-}{\rightleftarrows }} X_4+X_3 \qquad X_4 \overset{k_4^+}{\underset{k_4^-}{\rightleftarrows }} X_1 \end{aligned}$$

$$ J=\left[ \begin{array}{ccccc} -(k_1^++k_4^-) & 0 & 0 & k_4^+ & 0 \\ k_1^+ & -k_2^+ & 0 & 0 & k_2^- \\ k_1^+ & 0 & -k_3^+ & k_3^- & k_5^+ \\ k_4^- & 0 & k_3^+ & -(k_4^++k_3^-) & k_5^+ \\ 0 & k_2^+ & 0 & 0 & -(k_5^++k_2^-) \end{array} \right] $$

Which gives non-zero Det $J=2k_1^+k_2^+k_3^+k_4^+k_5^+$

#### Type V

7.4.6 $$\begin{aligned} X_1 \overset{k_1^+}{\underset{k_1^-}{\rightleftarrows }} X_2+X_3 \qquad X_2 \overset{k_2^+}{\underset{k_2^-}{\rightleftarrows }} X_4 \qquad X_3 \overset{k_3^+}{\underset{k_3^-}{\rightleftarrows }} X_5 \\ \nonumber X_4 \overset{k_4^+}{\underset{k_4^-}{\rightleftarrows }} X_6+X_3 \qquad X_5 \overset{k_5^+}{\underset{k_5^-}{\rightleftarrows }} X_6+X_2 \qquad X_6 \overset{k_6^+}{\underset{k_6^-}{\rightleftarrows }} X_1 \end{aligned}$$

$$ J=\left[ \begin{array}{cccccc} -(k_1^++k_6^-) & 0 & 0 & 0 & 0 & k_6^+ \\ k_1^+ & -k_2^+ & 0 & k_2^- & k_5^+ & 0 \\ k_1^+ & 0 & -k_3^+ & k_4^+ & k_3^- & 0 \\ 0 & k_2^+ & 0 & -(k_4^++k_2^-) & 0 & 0 \\ 0 & 0 & k_3^+ & 0 & -(k_5^++k_3^-) & 0 \\ k_6^- & 0 & 0 & k_4^+ & k_5^+ & -k_6^+ \end{array} \right] $$

Which computes to Det $J=-4k_1^+k_2^+k_3^+k_4^+k_5^+k_6^+$, non-zero
