# Supplementary material for: Autocatalytic cores in the diluted regime: classification and properties
Source: J Math Biol. 2026 Feb 21;92(3):36. doi: 10.1007/s00285-026-02357-7 (PMC12924792; doi:10.1007/s00285-026-02357-7)
Supplement: Supplementary file 1 — (pdf 341 KB) [file 285_2026_2357_MOESM1_ESM.pdf]

Supplementary Information: Autocatalytic cores in the diluted regime: classification and properties

December 9, 2025

Praneet Nandan[a], Philippe Nghe[a,b] and Jérémie Unterberger[c]

[a]Laboratory of Biophysics and Evolution,[1] ESPCI Paris-PSL,
10 Rue Vauquelin, 75005 Paris, France
[b]Laboratoire de Biologie Structurale de la Cellule (BIOC), CNRS, Ecole polytechnique,
Institut Polytechnique de Paris,91120 Palaiseau , France
[c]Institut Elie Cartan,[2] Université de Lorraine,
B.P. 239, F – 54506 Vandœuvre-lès-Nancy Cedex, France
praneet.nandan@espci.fr

[1]UMR CNRS-ESPCI 8231 Chemistry, Biology and Innovation
[2]Laboratoire associé au CNRS UMR 7502.

Contents

S1 Proof of Classification Theorem **2**
S1.1 Case 1 (simple reactions) 3
S1.2 Case 2 (one cycle) 3
S1.3 Two one-to-many reactions 5
S1.4 Three one-to-many reactions, different arks 8
S1.5 Four one-to-many reactions, different arks 10
S1.6 Left over case (see end of S1.3) 11

S2 Explicit formulas for the equilibrium state **12**

S3 General analysis of stationary states of cores (Types I and III) **15**

S4 General analysis of stationary states of cores (Type IV,II$_2$, and V) **17**
S4.1 Type IV 18
S4.2 Type II, ℓ=2 22
S4.3 Some degradations zero and some non-zero for Type II and Type IV 26
S4.4 Type V 26

S1 Proof of Classification Theorem

Let $G = (\mathcal{X}, \mathcal{R})$ be an autocatalytic core composed uniquely of one-to-one or one-to-many reactions. In particular, by Proposition 2.9 in the main text, it verifies (Top). *We first prove by absurd that G must be irreducible.* Namely, assume G contains two strongly connected components $\mathcal{C}_1, \mathcal{C}_2$ with $\mathcal{C}_1 \to \mathcal{C}_2$ and $\mathcal{C}_1$ minimal, i.e. such that $\nexists \mathcal{C}$, $\mathcal{C} \to \mathcal{C}_1$. (Top) implies the existence of a one-to-many reaction $R : X \to s_1 x_1 + \cdots + s_k x_k$ with $X, x_1, \ldots, x_j \in \mathcal{C}_1$ $(1 \le j \le k)$; $x_\ell \notin \mathcal{C}_1$ if $\ell > j$; and $s_1 + \ldots + s_j \ge 2$. Then the irreducible restricted network $G' = (\mathcal{C}_1, \mathcal{R}\Big|_{\mathcal{C}_1})$ (see Appendix) includes the one-to-many reaction $R\Big|_{\mathcal{C}_1} : X \to s_1 x_1 + \ldots + s_j x_j$, hence G' satisfies (Top). Therefore, G cannot be minimal.

This has the following significant consequence: *verifying (Top) for an autocatalytic core reduces to the trivial task of checking that the reaction set $\mathcal{R}$ contains a one-to-many reaction.* This is the key principle of the proof.

Going further, we consider mutually exclusive cases. We will often make use of the following remark. Let $G = (\mathcal{X}, \mathcal{R})$ be an irreducible autocatalytic network, $\mathcal{R}' \subset \mathcal{R}$ and $\mathcal{X}' \subset \mathcal{X}$ such that $(\mathcal{X}', \mathcal{R}') \neq (\mathcal{X}, \mathcal{R})$, i.e. either $\mathcal{R}' \subsetneq \mathcal{R}$ or $\mathcal{X}' \subsetneq \mathcal{X}$ (or both). Then

the restricted network $G' = (\mathcal{X}, \mathcal{R}')\Big|_{\mathcal{X}'}$ is autocatalytic if it is irreducible and contains a one-to-many reaction. This gives an easy argument to prove that G is not minimal.

A key notion in the classification is that of a cycle. Briefly said, a cycle

$$\mathcal{C}: \qquad x_1 \to x_2 \to \cdots \to x_n \to x_1 \tag{S1.1}$$

is a succession of split reactions $x_i \to x_{i+1}$ $(1 \le i \le n-1)$, $x_n \to x_1$ coming from reactions in $\mathcal{R}$. The (index) ordering $1 \prec 2 \prec \cdots \prec n \prec 1$ is defined along the cycle. It is invariant under cyclic permutations so that $k \prec \cdots \prec n \prec 1 \prec \cdots \prec k$ defines the same cyclic order.

S1.1 Case 1 (simple reactions)

We assume here that all reactions are simple(that is, either a one-to-one reaction or a one-to-many reaction with a single type of product species). By (Top), at least one of them is one-to-many, $R_1 : x_1 \to sx_2$ with $s \ge 2$. Since G is irreducible, one can find a cycle

$$\mathcal{C}: \qquad x_1 \to x_2 \to \cdots \to x_n \to x_1, \tag{S1.2}$$

Call $R_i : x_i \to s_i x_{i+1}$, $i = 1, \ldots, n$ the reactions along the cycle. Then $G' = (\mathcal{C}, \{R_1, \ldots, R_n\}) \subset G$ satisfies (Top), so (by minimality) $G = G'$ is of Type I.

S1.2 Case 2 (one cycle)

Barring Case 1., G contains at least one one-to-many reaction

$$R_1: \qquad x_1 \to s_2 x_2 + s' x' + \cdots \tag{S1.3}$$

We assume here that there exists a cycle $\mathcal{C} : x_1 \to x_2 \to \cdots \to x_n \to x_1$ containing all species.

It is easy to see that R_1 has exactly two different products; indeed, assuming (by absurd) $R_1 :\ x_1 \to s_2 x_2 + s' x' + s'' x'' + \cdots$, with $x_1 \prec x_2 \prec x' \prec x''$, the restricted network $G|_{\mathcal{X}'}$, $\mathcal{X}' = \{x' \to \cdots \to x_1\} \subsetneq \mathcal{X}$ (in blue) would also be irreducible and satisfy (Top), see Figure (the dotted line stands for possible extra split reactions $x_1 \to \cdots$ coming from R_1),

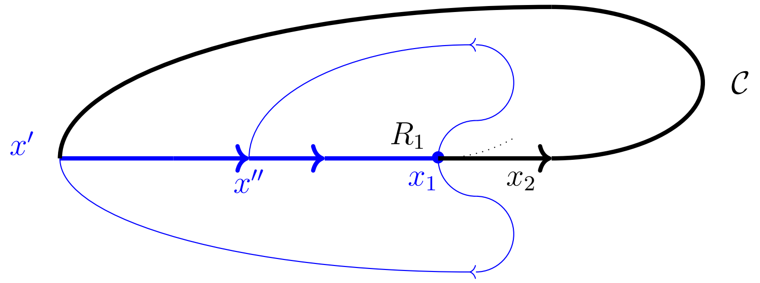

For the same reason, any reaction with ≥ 3 different products is excluded.

We note certain reactions that become redundant in this framework and can be removed without breaking (Top)

- **short-cuts**, i.e. simple reactions $x_i \to sx_j$ not along the cycle, i.e. such that $|j-i| \neq 1, n-1$
- $x_i \to s''x_{i+1}$ become redundant in presence of multiple reactions $x_i \to sx_{i+1} + s'x'$
- multiple reactions $x \to s'x' + s''x''$, such that neither of its split reactions $x \to x'$ nor $x \to x''$ is along the cycle
- reaction $x_i \to s_1 x_{i+1} + s'_1 x'_1$ is redundant in the presence of $x_i \to s_2 x_{i+1} + s'_2 x'_2$ (i.e. with different stoichiometric coefficients, $s_1 \neq s_2$ or $s'_1 \neq s'_2$, or different products, $x'_1 \neq x'_2$)

On the other hand, split reactions $x_i \to x_{i+1}$ may either be simple reactions, or a piece of (i.e. a split reaction coming from) a multiple reaction. Denote by $I_\ell = \{i_1, \ldots, i_\ell\}$ the set of indices i such that G contains a one-to-many reaction $R_i : x_i \to sx + s'x'$ with $x = x_{i+1}$. Then the multiple reactions of G belong to the closed list,

$$R_j : \qquad x_{i_j} \to s_{i_j} x_{i_j+1} + s'_j x_{\sigma_j}, \qquad j = 1, \ldots, \ell \tag{S1.4}$$

with $\sigma_j \in \{1, \ldots, n\}$, $\sigma_j \neq i_j, i_j + 1$. Denote by Σ_ℓ the set of these σ_j. See illustrating Figure corresponding to Type II in the Theorem in §??. The stoichiometric coefficient s'_j attached to the side-branch $j = 1, \ldots, \ell$, see (S1.4), is equal to 1 (otherwise $G\Big|_{\mathcal{C}_j}$ restricted to the subcycle $\mathcal{C}_j : x_{\sigma_j} \to x_{\sigma_j+1} \to \cdots \to x_{i_j} \to x_{\sigma_j}$ would be irreducible and satisfy (Top)).

We include $R_1, R_2, \ldots, R_\ell$ one after the another, and discuss by induction on $j \geq 2$ the various possibilities of inserting i_j, σ_j along the $2(j-1)$ arks defined by the previously defined indices $I_{j-1} \uplus \Sigma_{j-1} := \{i_1, \sigma_1, i_2, \sigma_2, \ldots, i_{j-1}, \sigma_{j-1}\}$. Namely – starting from i_1, following along the cycle, and considering all indices in $I_{j-1} \uplus \Sigma_{j-1}$ – we get

$$i_1 = \tau_1 \prec \cdots \prec \tau_{2(j-1)} \prec i_1 \tag{S1.5}$$

with $\{\tau_1, \ldots, \tau_{2(j-1)}\} = I_{j-1} \uplus \Sigma_{j-1}$. The $2(j-1)$ arks $(\square_i)_{1 \leq i \leq 2(j-1)}$ are then

$$\square_i = \{k \mid \tau_i \prec k \prec \tau_{i+1}\} \ \ (1 \leq i < 2(j-1)), \qquad \square_{2(j-1)} = \{k \mid \tau_{2(j-1)} \prec k \prec \tau_1\}. \tag{S1.6}$$

Note that some of them are possibly empty.

If $\ell = 1$, we get a type II cycle with ℓ=1.

Remark. Note that the indices of the reactions R_j, $j = \{1, 2, ...m\}$ are arbitrarily assigned; interchanging them does not change the type of cycle. This simple remark will be used many times.

S1.3 Two one-to-many reactions

We denote by

$$\square_1 = \{i \neq i_1, \sigma_1 \mid i_1 \prec i \prec \sigma_1\} \tag{S1.7}$$

the set of indices found along the ark from i_1 to σ_1, and similarly

$$\square_2 = \{i \neq i_1, \sigma_1 \mid \sigma_1 \prec i \prec i_1\} \tag{S1.8}$$

the complementary set. Thus

$$i_1 \prec \square_1 \prec \sigma_1 \prec \square_2 \tag{S1.9}$$

by which it is meant that $i_1 \prec i \prec \sigma_1 \prec i'$ for all $i \in \square_1, i' \in \square_2$.

Adding a second one-to-many reaction is equivalent to choosing i_2 and σ_2 in $\square_1 \uplus \square_2$, or in other words, to inserting i_2, σ_2 along the two corresponding arks. Exhausting the different possibilities – with, in particular, i_2, σ_2 inserted along the same ark or not –, we find the following cases:

(i) (nested violation, N_1 case) $i_1 \prec i_2 \prec \sigma_2 \prec \sigma_1$ (both i_2, σ_2 inserted along $\square_1$)

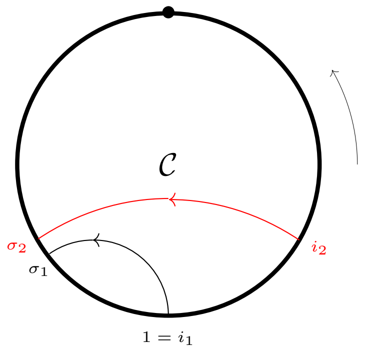

nested branches (excluded case)

Figure 1: Invalid nested branches

Actually, $\sigma_2 \neq i_2 + 1$ (as follows from (S1.4)) so that, more precisely, $i_1 \prec i_2 \prec\prec \sigma_2 \prec \sigma_1 \prec 1$. The black dot along the ark from i_2 to σ_2 indicates any of the species in the non-empty set of species $\mathcal{X}_{ext}$ indexed by $i_2 + 1, \ldots, \sigma_2 - 1$. Similarly, the notation $\prec\prec$ denotes the presence of at least one species between i_2 and σ_2, so that the newly formed ark $\mathcal{X}_{ext} = \{i_2 + 1 \prec \cdots \prec \sigma_2 - 1\}$ is not empty.

This is contradictory with minimality since the restricted network $G\Big|_{\mathcal{X}\setminus\mathcal{X}_{ext}}$ is strongly connected, and contains the one-to-many reaction R_1.

(ii) $i_1 \prec \sigma_1 \prec \sigma_2 \prec i_2$ or $i_1 \prec \sigma_1 \prec i_2 \prec \sigma_2$ (both i_2, σ_2 inserted along $\square_2$) is a nested violation case, as we can remove any species between i_1 and σ_1 and still get a strongly connected network with a one-to-many reaction (R_2).

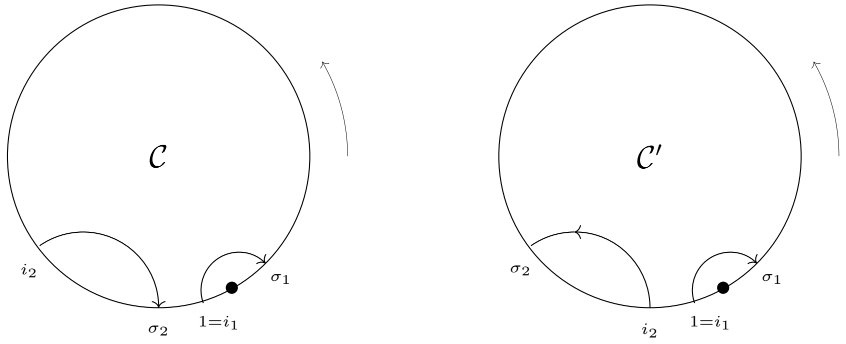

Whenever we get a form like $i_j \prec i_k \prec \sigma_k \prec \sigma_j$ (corresponding to one arc along the cycle) or $i_j \prec \sigma_j \prec i_k \prec \sigma_k$ (corresponding to two arcs along the cycle), we would henceforth refer to it as a nested violation or nested branches (N_1 and N_2 respectively).

(iii) $i_1 \prec \sigma_2 \prec i_2 \prec \sigma_1$ (i_2, σ_2 permuted compared to (i)) yields a Type II core with ℓ=2.

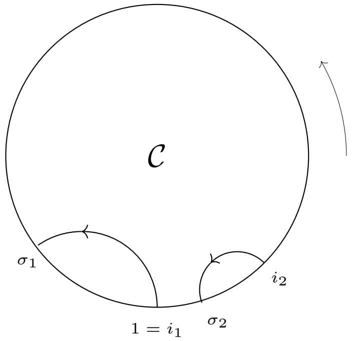

Type II

Figure 2: A cycle of Type II with ℓ=2

(iv) (entangled violation E case) $i_1 \prec i_2 \prec\prec \sigma_1 \prec \sigma_2$ (i_2 inserted along $\square_1$, σ_2 along $\square_2$, assuming further that $\sigma_1 \neq i_2 + 1$) We then reach the same conclusion as in (i) with $\mathcal{X}_{ext}$ indexed by $i_2 + 1, \ldots, \sigma_1 - 1$: the restricted network $G\Big|_{\mathcal{X}\setminus\mathcal{X}_{ext}}$ is strongly connected, and contains the one-to-many reaction R_1, therefore G is not minimal.

By extension, for any $j \geq 2$, we shall call **entangled violation** (of minimality) denoted by E, any pair configuration such that, for some pair of indices $1 \leq a \neq b \leq j$,

$$i_a \prec i_b \prec\prec \sigma_a \prec \sigma_b \qquad \text{(entangled violation)} \tag{S1.10}$$

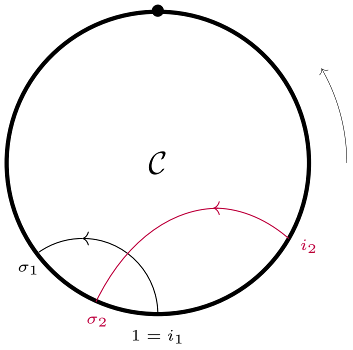

entangled branches (excluded case)

Remark. Nested configurations are sensitive to relative pair orientations, e.g. exchanging i_2 and σ_2 turns two nested branches (Fig. 1 above) into a Type II core with $\ell = 2$ (Fig. 2). So are entangled configurations, but note that

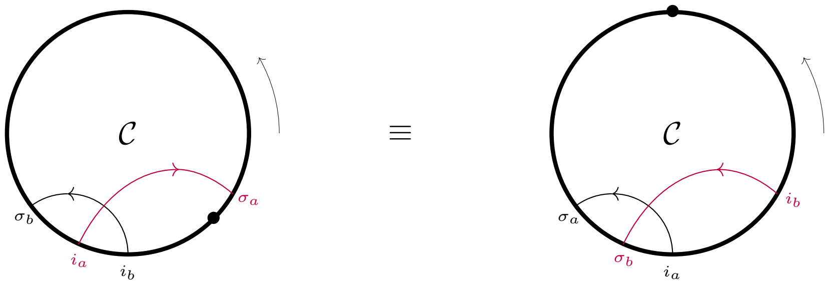

By moving around σ_a along the cycle, relative pair orientations of entangled pairs seem reversed (but the black dot, indicating that the ark from i_b to σ_a is not empty, is not at the same location).

(v) $i_1 \prec i_2 \prec \sigma_1 \prec \sigma_2$ as in (iv), but (special case) $\sigma_1 = i_2 + 1$. This is a Type IV core, with the identification $\{i_1 \prec i_2 \prec \sigma_1 = i_2 + 1 \prec \sigma_2 \prec i_1\} \leftrightarrow \{v \prec w \prec x' \prec u \prec v\}$ (the cycle containing all species is uncovered by dropping the edges $v \to x'$ and $w \to u$).

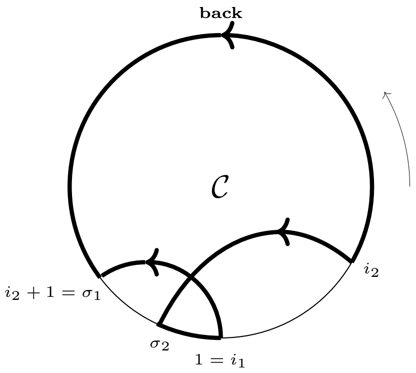

Type IV

(vi) $i_1 \prec \sigma_2 \prec \sigma_1 \prec i_2$ (i_2 inserted along $\square_2$, σ_2 along $\square_1$) Exchanging indices 1,2 yields back cases (iv)-(v).

In the following, we add inductively pairs of indices (i_j, σ_j), $j = 3, 4, \ldots$ For $j = 2$, we got only two cores (Types II and IV), all other possibilities being non-minimal autocatalytic. Clearly, adding another multiple reaction ($j = 3$) to a non-minimal autocatalytic network cannot yield a minimal autocatalytic network, so that we may content ourselves with starting from a Type II or Type IV core and adding a pair of indices (i_3, σ_3), discarding systematically nested and entangled violations.

It proves convenient to postpone the discussion of the relatively simple case when i_j and σ_j are located along the same ark (see §$S1.6$ below). So we assume for now that they belong to two different arks (a condition stipulated simply as 'different arks').

S1.4 Three one-to-many reactions, different arks

We start either from a Type II or a Type IV core and add a couple of indices (i_3, σ_3) located on two different arks.

A. Starting from Type II_2 (see Fig. 3). The ordering along the circle is:

$$i_1 \prec \square_1 \prec \sigma_2 \prec \square_2 \prec i_2 \prec \square_3 \prec \sigma_1 \prec \square_4 \prec i_1. \tag{S1.11}$$

We discuss the various possibilities, to conclude that no new cores can be formed:

(i) ($i_3 \in \square_1$) Then necessarily $\sigma_3 \in \square_4$ (otherwise $\sigma_3 \in \square_2$ or $\square_3$, and – discarding the pair (i_2, σ_2) – one gets nested branches). But then the pairs of indices (i_1, σ_1), (i_3, σ_3) make an entangled violation. Similarly if $i_3 \in \square_3$, by exchanging (i_1, σ_1) and (i_2, σ_2).

(ii) ($i_3 \in \square_2$) Then $\sigma_3 \in \square_1$ (if $\sigma_3 \in \square_3$, (i_1, σ_1) and (i_3, σ_3) form nested branches and if $\sigma_3 \in \square_4$, the same pair forms an entangled violation). But then the pairs of indices (i_2, σ_2) and (i_3, σ_3) make an entangled violation. Similarly if $i_3 \in \square_4$, by exchanging (i_1, σ_1) and (i_2, σ_2).

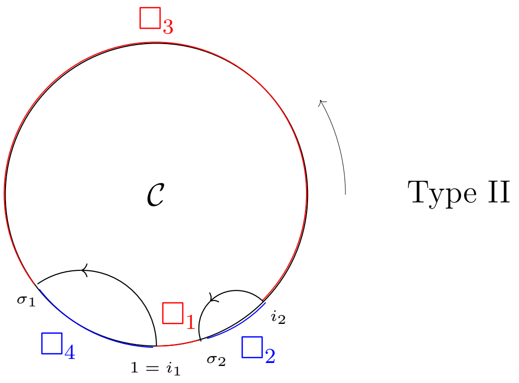

Figure 3: A cycle of Type II with ℓ=2 and arks

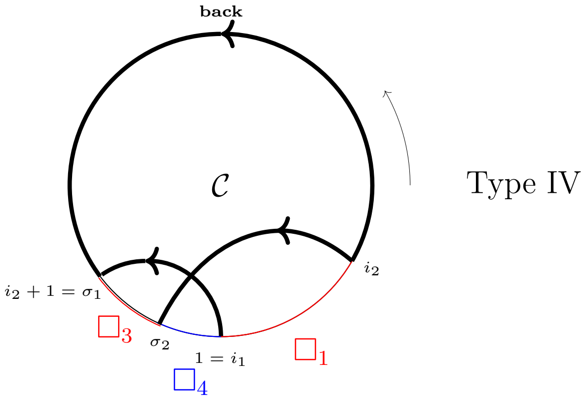

Figure 4: A cycle of Type IV with arks

B. Starting from Type IV (see Fig. 4). Since $\sigma_1 = i_2 + 1$, $\Box_2 = \emptyset$, and the ordering is now

$$i_1 \prec \Box_1 \prec i_2 \prec \sigma_1 \prec \Box_3 \prec \sigma_2 \prec \Box_4 \prec i_1 \tag{S1.12}$$

Discussing the various possibilities, we prove that only Type V cores can be formed this way:

(i) ($i_3 \in \Box_1$) If $\sigma_3 \in \Box_4$, the pairs (i_2, σ_2) and (i_3, σ_3) form nested branches. On the other hand, if $\sigma_3 \in \Box_3$, the same pairs form an entangled violation.

(ii) ($i_3 \in \Box_3$) If $\sigma_3 \in \Box_4$, then pairs (i_1, σ_1) and (i_3, σ_3) form a nested violation (N_2). Hence necessarily $\sigma_3 \in \Box_1$, so that

$$i_1 \prec \sigma_3 \prec i_2 \prec \sigma_1 \prec i_3 \prec \sigma_2. \tag{S1.13}$$

Here there is no outright violation of any case, but we note that the pairs (i_2, σ_2) and (i_3, σ_3), and also the pairs (i_1, σ_1) and (i_3, σ_3), take the form of Section 3.2.1 cases (iv)-(v). This means that we must have $\sigma_1 = i_2 + 1$ (from the 1,2 pairs), $\sigma_3 = i_1 + 1$ (from the 3,1 pairs) and $\sigma_2 = i_3 + 1$ (from the 2,3 pairs). This is a Type V core, with the identification $\{i_1 \prec \sigma_3 = i_1 + 1 \prec i_2 \prec \sigma_1 = i_2 + 1 \prec i_3 \prec \sigma_2 = i_3 + 1 \prec i_1\} \leftrightarrow \{v \prec x \prec w \prec x' \prec w' \prec u \prec v\}$. The cycle connecting all species is uncovered by dropping the edges $v \to x'$, $w \to u$ and the backbranch $w' \to x$.

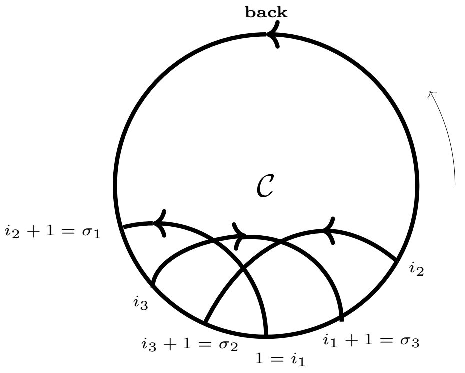

Type V

(iii) ($i_3 \in \Box_4$) If $\sigma_3 \in \Box_1$, pairs (i_2, σ_2) and (i_3, σ_3) form nested branches (N_2). On the other hand, if $\sigma_3 \in \Box_3$, these pairs form an entangled violation.

S1.5 Four one-to-many reactions, different arks

Starting from the Type V core produced in §S1.4 **B.** (ii), we add a new pair (i_4, σ_4) in different arks. The ordering along the cycle is now (see previous Figure)

$$i_1 \prec \sigma_3 = i_1 + 1 \prec \Box_2 \prec i_2 \prec \sigma_1 = i_2 + 1 \prec \Box_4 \prec i_3 \prec \sigma_2 = i_3 + 1 \prec \Box_6 \prec i_1 \quad \text{(S1.14)}$$

with $\Box_1, \Box_3, \Box_5 = \emptyset$. Discussing the various possibilities, we find that no new core can be formed this way:

(i) ($i_4 \in \Box_2$) If $\sigma_4 \in \Box_4$, the pairs (i_4, σ_4) and (i_3, σ_3) form a nested violation (N_2). If $\sigma_4 \in \Box_6$, then the pairs (i_2, σ_2) and (i_4, σ_4) form a nested violation (N_1).

(ii) ($i_4 \in \Box_4$) If $\sigma_4 \in \Box_2$, the pairs (i_4, σ_4) and (i_3, σ_3) form a nested violation (N_1). If $\sigma_4 \in \Box_6$, pairs (i_1, σ_1) and (i_4, σ_4) form a nested violation (N_2).

(iii) ($i_4 \in \Box_6$) If $\sigma_4 \in \Box_2$, then pairs (i_2, σ_2) and (i_4, σ_4) form a nested violation (N_2). If $\sigma_4 \in \Box_4$, then pairs (i_1, σ_1) and (i_4, σ_4) form a nested violation (N_1).

S1.6 Left over case (see end of S1.3)

The left over case is obtained starting from Type II or Type IV and inserting the pair i_j, σ_j in the same arc. This cannot be done in the ordered pair $i_j \prec \sigma_j$ (§S1.3 N_1). Thus it is done in the form of the ordered pair $\sigma_j \prec i_j$ (i.e. opposite in direction to the cycle).

For this insertion into $\Box_i$, a cyclic permutation of the indices will not result in N_1 violation only if starting from $\Box_i$, all σ_k (k< j) are encountered before their corresponding i_k.

- For type IV cycles (§S1.4), there is no such ark that can satisfy this condition.

- For type II with ℓ =2, where we had $i_1 \prec \Box_1 \prec \sigma_2 \prec \Box_2 \prec i_2 \prec \Box_3 \prec \sigma_1 \prec \Box_4$, we can insert this pair in $\Box_1$ or $\Box_3$ which will both yield the same notation except for an exchange of labels for reactions 2 and 3. We will get
  $i_1 \prec \sigma_2 \prec i_2 \prec \sigma_3 \prec i_3 \prec \sigma_1$
  Which is a Type II reaction with ℓ=3.

We can keep inserting such $\sigma_j \prec i_j$ pairs in such a notation right after any i_k, k<j, without violating the minimality condition. We get the general notation for the type II_ℓ cycle -
$i_1 \prec \sigma_2 \prec i_2 \prec \sigma_3 \prec i_3 \prec ..... \prec \sigma_\ell \prec i_\ell \prec \sigma_1$

If on the other hand we try inserting $\sigma_{\ell+1}$ and $i_{\ell+1}$ into this general notation at separate

places (for ℓ>2), we will always get a violation. This is shown below.

Assume on the contrary that you could insert $\sigma_{\ell+1}$ and $i_{\ell+1}$ in a cycle of type II$_\ell$, but not in the form $\sigma_{\ell+1} \prec i_{\ell+1}$ (or $i_{\ell+1} \prec \sigma_{\ell+1}$, which is always a nesting violation). This implies that there are necessarily other species in between $\sigma_{\ell+1}$ and $i_{\ell+1}$ and also between $i_{\ell+1}$ and $\sigma_{\ell+1}$. We select indices k1 and k2, such that either i_{k1} or σ_{k1} (but not both) is between $\sigma_{\ell+1}$ and $i_{\ell+1}$ and either i_{k2} or σ_{k2} (but not both) is between $i_{\ell+1}$ and $\sigma_{\ell+1}$ in the notation.

Then the loop takes the form:

$$i_{k1} \prec \square_1 \prec \sigma_{k2} \prec \square_2 \prec i_{k2} \prec \square_3 \prec \sigma_{k1} \prec \square_4 \prec i_{k1}. \tag{S1.15}$$

With $i_{\ell+1}$ and $\sigma_{\ell+1}$ filling up different squares. This is identical to §S1.4 Part 1 except for a change of index (there also, we had to insert an entangled branch in a Type II cycle). Since all the cases there were a violation of minimality (E violation), this case also violates minimality.

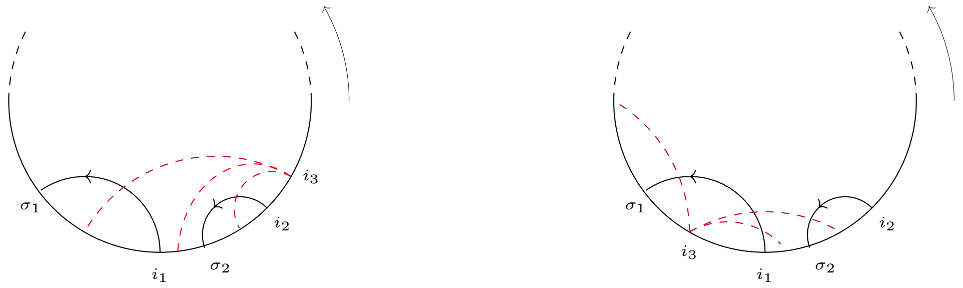

No entangled branches possible for type II ℓ> 1

Thus any minimal, strongly connected cycle with more than three one-to-many reactions has to be of Type II, and up to the case of Three one-to-many reactions we have shown that minimality is violated for a single cycle apart from the types discussed in the classification theorem.

S2 Explicit formulas for the equilibrium state

We use the classification theorem (Theorem 3.1 in main text) to give explicit formulas for 'minimal' stoichiometric coefficients (all coefficients equal to 0 or 1, except $s_n = 2$ for the replication reaction $x_n \to x_1$ in Type I).

(i) (Type I) Setting to zero the currents associated to the reactions $x_j \underset{k_j^-}{\overset{k_j^+}{\rightleftarrows}} x_{j+1}$, $j = 1, \ldots, n-1$ along the branch

$$x_1 \to x_2 \to \cdots \to x_n \tag{S2.1}$$

yields a sequence of equations $k_1^+ C_{stat,1} = k_1^- C_{stat,2}, \ldots, k_{n-1}^+ C_{stat,n-1} = k_{n-1}^- C_{stat,n}$ which define each $C_{stat,j}$, $j = 1, \ldots, n-1$ uniquely in terms of $C_{stat,n}$; in particular, $C_{stat,1} = (K(1 \rightleftarrows n))^{-1} C_{stat,n}$, where

$$K(1 \rightleftarrows n) = \frac{k_1^+ \cdots k_{n-1}^+}{k_{n-1}^- \cdots k_1^-} \tag{S2.2}$$

is the quotient (product of direct rates)/(product of reverse rates) along the branch. Completing the loop, we get $k_n^+ C_{stat,n} = k_n^- C_{stat,1}^2$. Substituting $K(1 \rightleftarrows n) C_{stat,1}$ to $C_{stat,n}$ yields

$$C_{stat,1} = K(1 \rightleftarrows n) \frac{k_n^+}{k_n^-}, \qquad C_{stat,k} = K(1 \rightleftarrows k) C_{stat,1} \ \ (2 \le k \le n) \tag{S2.3}$$

(ii) (Type II) Following each subcycle $\mathcal{C}_j : x_{\sigma_j} \to x_{\sigma_j+1} \to \cdots \to x_j$ $(j = 1, \ldots, \ell)$, one gets as in (i) $C_{stat,\sigma_j} = (K(\sigma_j \rightleftarrows j))^{-1} C_{stat,j}$. Then the current associated to $R_j : \ x_{i_j} \to x_{i_j+1} + x_{\sigma_j}$ vanishes if and only if $k_{i_j}^+ C_{stat,i_j} = k_{i_j}^- C_{stat,i_j+1} C_{stat,\sigma_j}$. This yields

$$C_{stat,i_j+1} = \frac{k_{i_j}^+}{k_{i_j}^-} \frac{C_{stat,i_j}}{C_{stat,\sigma_j}} = K(\sigma_j \rightleftarrows j) \frac{k_{i_j}^+}{k_{i_j}^-} \tag{S2.4}$$

and more generally,

$$C_{stat,m} = K(\sigma_j \rightleftarrows m), \qquad i_j \prec m \preceq i_{j+1} \tag{S2.5}$$

with $K(m' \rightleftarrows m) = \frac{k_{m'}^+ \cdots k_m^+}{k_m^- \cdots k_{m'}^-}$ is the quotient (product of direct rates)/(product of reverse rates) along the subcycle $m' \to m'+1 \to \cdots m$.

(iii) (Type III) Removing first the one-to-many reaction $R_v : \ v \underset{k_v^-}{\overset{k_v^+}{\rightleftarrows}} s_v x + s'_v x'$ (and the associated reverse reaction), one gets a star-shaped network, with one branch starting from u and ending in v, and two branches starting from x, resp. x' and ending in u. Proceeding as in (i) along each of these branches, one gets the relations

$$C_{stat,u} = (K(u \rightleftarrows v))^{-1} C_{stat,v}, \qquad C_{stat,x} = (K(x \rightleftarrows u))^{-1} C_{stat,u}, \qquad C_{stat,x'} = (K(x' \rightleftarrows u))^{-1} C_{stat,u}. \tag{S2.6}$$

Now, the current associated to R_v vanishes if and only if $k_v^+ C_v = k_v^- C_x C_{x'}$. Using (S2.6) yields

$$C_{stat,u} = K(u \rightleftarrows v)K(x \rightleftarrows u)K(x' \rightleftarrows u)\frac{k_v^+}{k_v^-}$$
$$C_{stat,x} = K(u \rightleftarrows v)K(x' \rightleftarrows u)\frac{k_v^+}{k_v^-}, \qquad C_{stat,x'} = K(u \rightleftarrows v)K(x \rightleftarrows u)\frac{k_v^+}{k_v^-} \tag{S2.7}$$

from which the other coefficients $C_{stat,\cdot}$ are easily determined.

(iv) (Type IV) We proceed as in (iii) by removing first the one-to-many reactions R_v and $R_w :\ w \underset{k_w^-}{\overset{k_w^+}{\rightleftarrows}} u + x'$. Then we have one branch from x' to v through u along $\mathcal{C}'$, and another branch from x to w along $\mathcal{C}$, from which we get the relations

$$C_{stat,x'} = (K(x' \rightleftarrows v))^{-1} C_{stat,v},\ C_{stat,u} = (K(u \rightleftarrows v))^{-1} C_{stat,v},\ C_{stat,x} = (K(x \rightleftarrows w))^{-1} C_{stat,w} \tag{S2.8}$$

Now, the currents associated to R_v, R_w vanish if and only if

$$k_v^+ C_{stat,v} = k_v^- C_{stat,x} C_{stat,x'}, \qquad k_w^+ C_{stat,w} = k_w^- C_{stat,u} C_{stat,x'}. \tag{S2.9}$$

Substituting (S2.8) into the previous relations yields

$$C_{stat,w} = K(x \rightleftarrows w)K(x' \rightleftarrows v)\frac{k_v^+}{k_v^-}, \qquad C_{stat,x} = K(x' \rightleftarrows v)\frac{k_v^+}{k_v^-}$$
$$C_{stat,v} = K(x' \rightleftarrows v)\sqrt{K(x \rightleftarrows w)K(u \rightleftarrows v)\frac{k_w^+}{k_w^-}\frac{k_v^+}{k_v^-}} \tag{S2.10}$$

from which the other coefficients $C_{stat,\cdot}$ are easily determined.

(v) (Type V) We proceed as in (iv) by first removing the one-to-many reactions R_v, R_w and $R_{w'} :\ w' \underset{k_{w'}^-}{\overset{k_{w'}^+}{\rightleftarrows}} u + x$. Then we have one branch from x' to w' along $\mathcal{C}'$, one branch from x to w along $\mathcal{C}$, and one branch from u to v along $\mathcal{C} \cap \mathcal{C}'$, from which we get the relations

$$C_{stat,x'} = (K(x' \rightleftarrows w'))^{-1} C_{stat,w'},\ C_{stat,u} = (K(u \rightleftarrows v))^{-1} C_{stat,v},\ C_{stat,x} = (K(x \rightleftarrows w))^{-1} C_{stat,w} \tag{S2.11}$$

The currents associated to $R_v, R_w, R_{w'}$ vanish if and only if

$$k_v^+ C_{stat,v} = k_v^- C_{stat,x} C_{stat,x'}, \qquad k_w^+ C_{stat,w} = k_w^- C_{stat,u} C_{stat,x'}, \qquad k_{w'}^+ C_{stat,w'} = k_{w'}^- C_{stat,u} C_{stat,x} \tag{S2.12}$$

Substituting (S2.11) into the previous relations yields

$$(1) \qquad C_{stat,v} = \frac{k_v^-/k_v^+}{K(x \rightleftarrows w)K(x' \rightleftarrows w')}\, c_w^* c_{w'}^*,$$

$$(2) \qquad C_{stat,w} = \frac{k_w^-/k_w^+}{K(u \rightleftarrows v)K(x' \rightleftarrows w')}\, c_v^* c_{w'}^*, \qquad (3) \qquad C_{stat,w'} = \frac{k_{w'}^-/k_{w'}^+}{K(u \rightleftarrows v)K(x \rightleftarrows w)}\, c_v^* c_w^* \tag{S2.13}$$

Forming quotients $\frac{(1)}{(2)}, \frac{(1)}{(3)}$,

$$\frac{C_{stat,v}}{C_{stat,w}} = \sqrt{\frac{k_v^-}{k_v^+}\frac{k_w^+}{k_w^-}\frac{K(u \rightleftarrows v)}{K(x \rightleftarrows w)}}, \qquad \frac{C_{stat,v}}{C_{stat,w'}} = \sqrt{\frac{k_v^-}{k_v^+}\frac{k_{w'}^+}{k_{w'}^-}\frac{K(u \rightleftarrows v)}{K(x' \rightleftarrows w')}} \tag{S2.14}$$

makes it possible to eliminate $C_{stat,w}, C_{stat,w'}$ from (1), giving finally

$$C_{stat,v} = K(u \rightleftarrows v)\sqrt{K(x \rightleftarrows w)K(x' \rightleftarrows w')\frac{k_w^+}{k_w^-}\frac{k_{w'}^+}{k_{w'}^-}} \tag{S2.15}$$

and similar formulas for $C_{stat,w}, C_{stat,w'}$ by circular permutations between v, w, w'.

S3 General analysis of stationary states of cores (Types I and III)

General notations.

1. Let cyc be one of the cycles ($\mathcal{C}$ for Types I-II, $\mathcal{C}$ or $\mathcal{C}'$ for Types III-V). Assume $x \in$cyc is not the reactant/product of a multiple reaction, then x_+, resp. x_- is the species following, resp. preceding x along cyc. The rate of the reactions connecting $x_\pm$ to x are $x_- \overset{k_{x_-}^+}{\underset{k_{x_-}^-}{\rightleftarrows}} x$ and $x \overset{k_x^+}{\underset{k_x^-}{\rightleftarrows}} x_+$.

2. Let $R : x \to sz + s'z'$ be a multiple reaction with two different products z, z'. Then rates are $x \overset{\nu_x^+}{\underset{\nu_x^-}{\rightleftarrows}} sz + s'z'$.

Type I. The stationary equation for B_n yields the "linearization" equation,

$$(\nu_+ + k^-_{n-1} + a_n)[B_n] - k^+_{n-1}[B_{n-1}] = \nu_-[B]^2 \tag{S3.1}$$

Substituting into the equation for $B = B_0$ yields

$$(k_{on} + a_0)[B] - k_{off}[B_1] - 2k^+_{n-1}[B_{n-1}] = -2(k^-_{n-1} + a_n)[B_n] \tag{S3.2}$$

a Markov-type equation for B, with Markov transitions from B_1 and (shunting B_n) from B_{n-1}. Then the equations for $B_i, i = 1, \ldots, n-1$ describe Markov transitions to and from $B_{i\pm1}$, including the special case $i = n-1$,

$$(k^+_{n-1} + k^-_{n-2} + a_{n-1})[B_{n-1}] - k^+_{n-2}[B_{n-2}] = k^-_{n-1}[B_n] \tag{S3.3}$$

All together, we get

$$-\underline{M}\left([B]\ [B_1]\ \cdots\ [B_{n-1}]\right)^t = [B_n]\phi \tag{S3.4}$$

where $\underline{M}$ is a generalized irreducible Markov matrix describing transitions along the shortened cycle $B_0 \leftrightarrow B_1 \leftrightarrow \cdots \leftrightarrow B_{n-1}$ (with an arc $B_{n-1} \xrightarrow{2k^+_{n-1}} B_0$) with positive killing rates $a_1, \ldots, a_n$, and $\phi = \begin{pmatrix} \phi_1 \\ \vdots \\ \phi_n \end{pmatrix}$. The solution of (S3.4) is unique,

$$[B] = \psi_0[B_n], \qquad [B_i] = \psi_i[B_n], \qquad i = 1, \ldots, n-1 \tag{S3.5}$$

We must assume $\psi_i > 0$. Putting back these expressions for $[B], [B_{n-1}]$ into (S3.1) yields finally

$$\nu_-\psi_0^2[B_n] = (\nu_+ + k^-_{n-1} + a_n) - k^+_{n-1}\psi_{n-1}. \tag{S3.6}$$

If $\psi_i > 0$, $i = 1, \ldots, n-1$ and $(\nu_+ + k^-_{n-1} + a_n) > k^+_{n-1}\psi_{n-1}$, these formulas yield a unique stationary state.

Type III. The stationary equation for A_n yields the "linearization" equation,

$$(\nu_+ + k^-_{n-1} + a_n)[A_n] - k^+_{n-1}[A_{n-1}] = \nu_-[B'_0][B''_0] \tag{S3.7}$$

Proceeding as for Type I, we want to shunt $[A_n]$. Putting aside the equation for A_n, we have $|\mathcal{X}| - 1$ Markov-type equations for the other species; specific cases are the equations for A_{n-1},

$$(k^+_{n-1} + k^-_{n-2} + a_{n-1})[A_{n-1}] - k^+_{n-2}[A_{n-2}] = k^-_{n-1}[A_n] \tag{S3.8}$$

and the equations for B'_0, B''_0, which we linearize using (S3.7),

$$(k'_{0,+} + a'_0)[B'_0] - k'_{0,-}[B'_1] - k^+_{n-1}[A_{n-1}] = -(k^-_{n-1} + a_n)[A_n] \tag{S3.9}$$

and similarly with $' \to ''$. We thus get a linear equation of the type

$$-\underline{M}([A_0] \ \cdots \ [A_{n-1}] \ [B'_0] \ \cdots \ [B'_n] \ [B''_0] \ \cdots \ [B''_n])^t = [A_n]\phi \tag{S3.10}$$

where $\underline{M}$ is a generalized irreducible Markov matrix describing transitions along the shortened cycle

$$\begin{array}{l} B'_n \leftrightarrow B'_n \leftrightarrow \cdots \leftrightarrow B'_0 \\ \updownarrow \qquad\qquad\qquad\quad \nwarrow^{k^+_{n-1}} \\ A_0 \leftrightarrow B_1 \leftrightarrow \cdots \leftrightarrow A_{n-1} \\ \updownarrow \qquad\qquad\qquad\quad \swarrow^{k^+_{n-1}} \\ B''_n \leftrightarrow B''_n \leftrightarrow \cdots \leftrightarrow B''_0 \end{array}$$

with positive killing rates. The unique solution is of the form

$$[A_i] = \psi_i[A_n],\ 0 \le i \le n-1; \qquad [B'_i] = \psi'_i[A_n],\ 0 \le i \le n'; \qquad [B''_i] = \psi''_i[A_n],\ 0 \le i \le n'' \tag{S3.11}$$

Inserting this into (S3.7) yields

$$\nu_- \psi'_0 \psi''_0 [A_n] = (\nu_+ + k^-_{n-1} + a_n) - k^+_{n-1}\psi_{n-1}. \tag{S3.12}$$

As in the case of Type I, if all coefficients $\psi_i, \psi'_i, \psi''_i$ are > 0 and $(\nu_+ + k^-_{n-1} + a_n) > k^+_{n-1}\psi_{n-1}$, these formulas define a unique stationary state.

S4 General analysis of stationary states of cores (Type IV,II$_2$, and V)

Lemma S4.1. *Species not involved in splitting reactions can be linearly substituted at the stationary state into the mass action equations of other species without affecting the sign of coefficients in that equation*

Proof. Consider a chunk of any Type of core of the form:

$$\rightleftarrows X_{j-1} \underset{k^-_{j-1}}{\overset{k^+_{j-1}}{\rightleftarrows}} X_j \underset{k^-_j}{\overset{k^+_j}{\rightleftarrows}} X_{j+1} \rightleftarrows$$

If the mass action system of equations are denoted by $f_i(x \in \mathcal{X})$ for i=1, 2...n,

$$f_j = -(k^+_j + k^-_{j-1} + a_j)[x_j] + k^-_j[x_{j+1}] + k^+_{j-1}[x_{j-1}] \tag{S4.1}$$

At the stationary state, $f_j = 0$ yields $[x_j]$ as a linear combination of $[x_{j-1}]$ and $[x_{j+1}]$, which can be used to write the flow between X_{j-1} and X_j as it appears in f_{j-1} in terms of $[x_{j+1}]$,

$$-k^+_{j-1}[x_{j-1}] + k^-_{j-1}[x_j] = -k^+_{j-1}[x_{j-1}] + \frac{k^-_{j-1}k^-_j[x_{j+1}]}{k^+_j + k^-_{j-1} + a_j} + \frac{k^-_{j-1}k^+_{j-1}[x_{j-1}]}{k^+_j + k^-_{j-1} + a_j} \tag{S4.2}$$

where we can note that the coefficient of $[x_{j-1}]$ remains negative while the $[x_j]$ with a positive coefficient is replaced by $[x_{j+1}]$ with a positive coefficient.

$$\frac{k^-_{j-1}k^-_j[x_{j+1}]}{k^+_j + k^-_{j-1} + a_j} - \frac{k^+_j k^+_{j-1}[x_{j-1}]}{k^+_j + k^-_{j-1} + a_j} - \frac{a_j k^+_{j-1}[x_{j-1}]}{k^+_j + k^-_{j-1} + a_j} = \bar{k}^-_{j-1}[x_{j+1}] - \bar{k}^+_{j-1}[x_{j-1}] - \bar{a}_j[x_{j-1}] \tag{S4.3}$$

The latter term in $\bar{a}$ can be absorbed into the degradation of species $[x_{j-1}]$ itself, and the same rates (but with the opposite flow) appear in the equation for $[x_{j+1}]$. Thus X_j can be shorted out of the network.

S4.1 Type IV

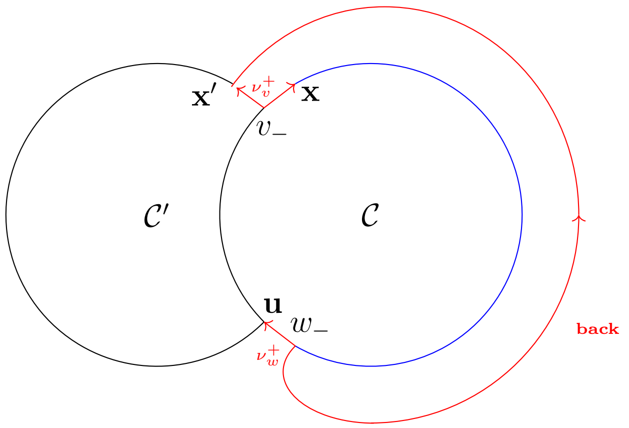

$\mathcal{X} = \{x, x', v, w, u\}$
We introduce two new variables δ and δ' as follows

$$\delta = \nu^+_v[v] - \nu^-_v[x][x'], \delta' = \nu^+_w[w] - \nu^-_w[u][x'] \tag{S4.4}$$

Then, using these variables and using the lemma on all species not involved in forks, we

get the mass action equations at stationarity as

$$\begin{aligned} -(k_x^+ + a_x)[x] + \delta + k_x^-[w] &= 0 \\ -(k_{x'}^+ + a_{x'})[x'] + \delta + \delta' + k_{x'}^-[u] &= 0 \\ -(k_u^+ + k_{x'}^- + a_u)[u] + \delta' + k_u^-[v] + k_{x'}^+[x'] &= 0 \\ -(k_u^- + a_v)[v] - \delta + k_u^+[u] &= 0 \\ -(k_x^- + a_w)[w] - \delta' + k_x^+[x] &= 0 \end{aligned} \tag{S4.5}$$

Further, from (S4.5) we also get that

$$2\delta' = a_{x'}[x'] + a_u[u] + a_v[v] \qquad \delta - \delta' = a_x[x] + a_w[w] \tag{S4.6}$$

which implies that for positive concentrations, δ and δ' are positive (i.e. the solution is in a subset of the positive quadrant)

Lemma S4.2. *For type IV core with rate constants and degradation values as in (S4.5), there exists a continuous function $m_b(a, k)$, such that any positive stationary state satisfies $m_b(a, k) \geq \max\{[z], z \in \mathcal{X}\}$*

Proof. The fact that δ and $\delta' \geq 0$ gives inequalities on the steady-state concentrations based on the rate constants from equation (S4.5),

$$\begin{aligned} &\frac{k_x^+ + a_x}{k_x^-}[x] \geq [w] \qquad \frac{k_{x'}^+ + a_{x'}}{k_{x'}^-}[x'] \geq [u] \\ &\frac{k_u^+ + k_{x'}^- + a_u}{k_u^-}[u] \geq [v] \qquad \frac{k_u^+ + k_{x'}^- + a_u}{k_{x'}^+}[u] \geq [x'] \end{aligned} \tag{S4.7}$$

As $\delta \geq 0$,

$$\left(\frac{k_{x'}^+ + a_{x'}}{k_{x'}^-}\right)\left(\frac{k_u^+ + k_{x'}^- + a_u}{k_u^-}\right)\nu_v^+[x'] \geq \nu_v^+[v] \geq \nu_v^-[x][x'] \tag{S4.8}$$

which for any non-zero stationary state gives

$$\left(\frac{k_{x'}^+ + a_{x'}}{k_{x'}^-}\right)\left(\frac{k_u^+ + k_{x'}^- + a_u}{k_u^-}\right)\frac{\nu_v^+}{\nu_v^-} \geq [x] \text{ (which also bounds } [w]) \tag{S4.9}$$

$\delta' \geq 0$ gives,

$$\nu_w^+[w] \geq \nu_w^-[u][x'] \geq \nu_w^-\left(\frac{k_{x'}^-}{k_{x'}^+ + a_{x'}}\right)[u]^2 \tag{S4.10}$$

And with the bound for $[w]$, we get an upper bound for $[u], [v]$ and $[x']$ as well. These bounds are the smallest when all the degradation is 0.

These equations can then be used to write down each of the concentrations $[x]$, $[x']$, $[u]$, $[v]$ and $[w]$ as a linear combination of δ and δ' with coefficients formed as expressions in the rate constants.

$$
\begin{aligned}
[x] &= \frac{(a_w + k_x^-)\delta - k_x^-\delta'}{a_w a_x + a_x k_x^- + a_w k_x^+} \qquad [w] = \frac{-(a_x + k_x^+)\delta' + k_x^+\delta}{a_w a_x + a_x k_x^- + a_w k_x^+} \\
[u] &= \frac{((a_v + k_u^-)a_{x'} + 2(a_v + k_u^-)k_{x'}^+)\delta' + (-a_{x'}k_u^- + a_v k_{x'}^+)\delta}{(a_v + k_u^-)a_{x'}k_{x'}^- + (a_u a_v + a_u k_u^- + a_v k_u^+)a_{x'} + (a_u a_v + a_u k_u^- + a_v k_u^+)k_{x'}^+} \\
[v] &= \frac{(a_{x'}k_u^+ + 2k_u^+ k_{x'}^+)\delta' - (a_u a_{x'} + a_{x'}k_u^+ + a_{x'}k_{x'}^- + a_u k_{x'}^+)\delta}{(a_v + k_u^-)a_{x'}k_{x'}^- + (a_u a_v + a_u k_u^- + a_v k_u^+)a_{x'} + (a_u a_v + a_u k_u^- + a_v k_u^+)k_{x'}^+} \\
[x'] &= \frac{(2a_v k_{x'}^- + 2k_u^- k_{x'}^- + a_u a_v + a_u k_u^- + a_v k_u^+)\delta' + (a_u a_v + a_u k_u^- + a_v k_u^+ + a_v k_{x'}^-)\delta}{(a_v + k_u^-)a_{x'}k_{x'}^- + (a_u a_v + a_u k_u^- + a_v k_u^+)a_{x'} + (a_u a_v + a_u k_u^- + a_v k_u^+)k_{x'}^+}
\end{aligned}
\tag{S4.11}
$$

The condition that the rate constants are all positive (> 0) directly give a strict sign to the coefficients of δ and δ' in the expressions of concentrations except for the coefficient of δ in the expression of $[u]$. Let $\epsilon = (-a_{x'}k_u^- + a_v k_{x'}^+)$. We can then replace these expressions in equation (S4.4) and taking care only for the sign, we get two new equations:-

$$
\begin{aligned}
0 &= A\delta' - B\delta - C\delta\delta' + D\delta'^2 - E\delta^2 \\
0 &= -A'\delta' + B'\delta + C'\delta\delta' - D'\delta'^2 + E'\delta^2
\end{aligned}
\tag{S4.12}
$$

where A, B, C, D, E and A', B', C', D', E' are functions of the rates, and for positive rate constants, all of them have positive values except C' and E'. For these two,
$\epsilon > 0 \Leftrightarrow E' < 0 \Rightarrow C' < 0$

Also important is the fact that since we want the concentrations to be non-negative,

$$
\begin{aligned}
0 &\geq -\nu_v^-[x][x'] \\
0 &\geq -\nu_w^-[u][x']
\end{aligned}
\tag{S4.13}
$$

$$
\begin{aligned}
0 &\geq -C\delta\delta' + D\delta'^2 - E\delta^2 \\
0 &\geq +C'\delta\delta' - D'\delta'^2 + E'\delta^2
\end{aligned}
\tag{S4.14}
$$

The stationary states of the type 4 core correspond to the solutions of equation (S4.12) in the positive quadrant following the inequalities (S4.14).

Lemma S4.3. *There is no degenerate stationary state in the positive quadrant for (S4.12)*

Proof. Assume that (S4.12) has a degenerate stationary state in the positive quadrant (not at origin) satisfying the given inequality,
The Jacobian Matrix of the system at this point is

$$J = \begin{bmatrix} -B - 2E\delta - C\delta' & A + 2D\delta' - C\delta \\ B' + 2E'\delta + C'\delta' & -A' - 2D'\delta' + C'\delta \end{bmatrix}$$

The state being degenerate implies that the columns of this matrix are linearly dependent, so assume there exists μ such that: Column 1 + Column 2 $\times \mu \frac{\delta'}{\delta} = 0$. In terms of expressions, this gives

$$\begin{aligned} 0 &= -B\delta - 2E\delta^2 - C\delta'\delta + \mu(A\delta' + 2D\delta'^2 - C\delta\delta') \\ 0 &= B'\delta + C'\delta\delta' + 2E'\delta^2 + \mu(-A'\delta' - 2D'\delta'^2 + C'\delta\delta') \end{aligned} \quad \text{(S4.15)}$$

Using equation (S4.12) gives

$$\begin{aligned} A\delta' - B\delta &= (\mu - 1)(A\delta' + 2D\delta'^2 - C\delta\delta') = (\mu - 1)(B\delta + D\delta'^2 + E\delta^2) \\ B'\delta - A'\delta' &= (\mu - 1)(-A'\delta' - 2D'\delta'^2 + C'\delta\delta') = (\mu - 1)(-B'\delta - E'\delta^2 - D'\delta'^2) \end{aligned} \quad \text{(S4.16)}$$

From the inequality (S4.14), we have that the LHS of both equations are positive. As we have assumed the solution to be in the positive quadrant, the RHS of the first equation being positive implies that $\mu > 1$.
We immediately have $\mu < 1$ if E' is positive. If it is negative, C' is negative as well and we get $\mu < 1$ in both cases. Thus we end up in a contradiction.

Lemma S4.4. *For degradation of the form $a' = \{\alpha a_x, \alpha a_u, \alpha a_w, \alpha a_v, \alpha a_{x'}\}$, the stationary state at the origin has Jacobian determinant 0 for only one value of $\alpha \in \mathbb{R}^+$*

Proof. The origin is always a solution for the hyperbolae for any value of degradation rates. The Jacobian matrix at this stationary state is

$$J = \begin{bmatrix} -B & A \\ B' & -A' \end{bmatrix}$$

Assume fixed reaction constants ($\{k,\nu\}$), $a = \{a_x, a_u, a_w, a_v, a_{x'}\}$, and let us consider the same system with degradation rates of the form ($a' = \{\alpha a_x, \alpha a_u, \alpha a_w, \alpha a_v, \alpha a_{x'}\}$) where $\alpha > 0$.

We formulate the Jacobian determinant at origin as a function of α.
Using equation (S4.11),

$$J = \begin{bmatrix} -(\alpha\, g(k,\nu,a) + \alpha\, h(k,\nu,a) + \alpha^2 p(k,\nu,a) + \alpha^3 q(k,\nu,a)) & f(k,\nu) + \alpha\, g(k,\nu,a) \\ f'(k,\nu) & -(f'(k,\nu) + \alpha\, g'(k,\nu,a) + \alpha^2 h'(k,\nu,a)) \end{bmatrix}$$

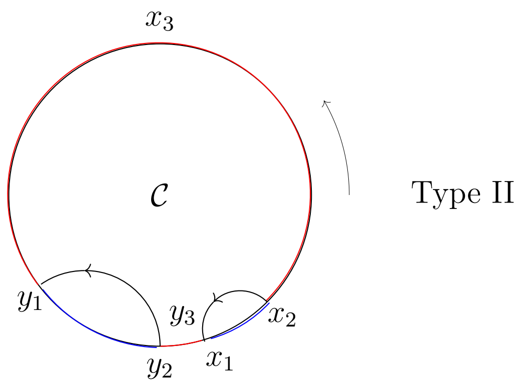

where f, g, h, p, q and f', g', h' are functions of the rate constants and degradation rates which have positive values for positive rates.

$$Det\ J = F(\alpha, k, \nu, a) - f(k, \nu) f'(k, \nu) \tag{S4.17}$$

where $F(\alpha, k, \nu, a)$ is, in fact, a degree 5 polynomial in α with all positive coefficients which is a strictly increasing function of α. For fixed rates and varying $\alpha > 0$, $Det\ J = 0$ for only one value of α.

Lemmas 8.3-5 imply that the Type IV cycle satisfies the assumptions of Theorem 5.1 (main text) if the zero stationary state is non-degenerate for vanishing degradation rates.

S4.2 Type II, ℓ=2

$\mathcal{X} = \{x_1, x_2, x_3, y_1, y_2, y_3\}$
We again introduce two new variables δ and δ' as follows

$$\delta = \nu_1^+[x_2] - \nu_1^-[x_3][x_1], \delta' = \nu_2^+[y_2] - \nu_2^-[y_3][y_1] \tag{S4.18}$$

Then, using these variables and using the lemma on all species not involved in forks, we get the mass action equations at stationary state as

$$\begin{aligned} -(k_{x_1}^+ + k_{y_3}^- + a_{x_1})[x_1] + \delta + k_{x_1}^-[x_2] + k_{y_3}^+[y_3] &= 0 \\ -(k_{x_1}^- + a_{x_2})[x_2] - \delta + k_{x_1}^+[x_1] &= 0 \\ -(k_{x_3}^+ + a_{x_3})[x_3] + \delta + k_{x_3}^-[y_1] &= 0 \\ -(k_{y_1}^+ + k_{x_3}^- + a_{y_1})[y_1] + \delta' + k_{y_1}^-[y_2] + k_{x_3}^+[x_3] &= 0 \\ -(k_{y_1}^- + a_{y_2})[y_2] - \delta' + k_{y_1}^+[y_1] &= 0 \\ -(k_{y_3}^+ + a_{y_3})[y_3] + \delta' + k_{y_3}^-[x_1] &= 0 \end{aligned} \tag{S4.19}$$

Further, from (S4.19) we also get that

$$\delta = a_{x_3}[x_3] + a_{y_1}[y_1] + a_{y_2}[y_2] \qquad \delta' = a_{y_3}[y_3] + a_{x_1}[x_1] + a_{x_2}[x_2] \tag{S4.20}$$

So again, we are restricted to the positive quadrant in the δ, δ' space.

Lemma S4.5. *For type II, $l = 2$ core with rate constants and degradation values as in (S4.19), there exists a continuous function $m_b(a,k)$, such that any positive stationary state satisfies $m_b(a,k) \geq \max\{[z], z \in \mathcal{X}\}$*

Proof. The inequalities after putting $\delta, \delta' \geq 0$ in (S4.19) are

$$\begin{aligned} &\frac{k_{x_1}^+ + k_{y_3}^- + a_{x_1}}{k_{x_1}^-}[x_1] \geq [x_2] \qquad \frac{k_{y_3}^+ + a_{y_3}}{k_{y_3}^-}[y_3] \geq [x_1] \\ &\frac{k_{y_1}^+ + k_{x_3}^- + a_{y_1}}{k_{y_1}^-}[y_1] \geq [y_2] \qquad \frac{k_{x_3}^+ + a_{x_3}}{k_{x_3}^-}[x_3] \geq [y_1] \end{aligned} \tag{S4.21}$$

And we get a bound for $[y_3]$ and $[x_3]$ from $\delta, \delta' \geq 0$ which bound everything

$$\begin{aligned} \left(\frac{k_{x_1}^+ + k_{y_3}^- + a_{x_1}}{k_{x_1}^-}\right)\nu_1^+[x_1] \geq \nu_1^+[x_2] \geq \nu_1^-[x_3][x_1] \\ \left(\frac{k_{y_1}^+ + k_{x_3}^- + a_{y_1}}{k_{y_1}^-}\right)\nu_2^+[y_1] \geq \nu_2^+[y_2] \geq \nu_1^-[y_3][y_1] \end{aligned} \tag{S4.22}$$

Concentrations in terms of these variables are

$$\begin{aligned}
[x_1] &= \frac{a_{x_2}(a_{y_3} + k_{y_3}^+)\delta + k_{y_3}^+(a_{x_2} + k_{x_1}^-)\delta'}{(a_{y_3}(a_{x_2} + k_{x_1}^-)k_{y_3}^- + (a_{x_1}a_{x_2} + a_{x_1}k_{x_1}^- + a_{x_2}k_{x_1}^+)a_{y_3} + (a_{x_1}a_{x_2} + a_{x_1}k_{x_1}^- + a_{x_2}k_{x_1}^+)k_{y_3}^+)} \quad \text{(S4.23)} \\
[x_2] &= \frac{k_{x_1}^+ k_{y_3}^+ \delta' - (a_{x_1}a_{y_3} + a_{y_3}k_{y_3}^- + a_{x_1}k_{y_3}^+)\delta}{(a_{y_3}(a_{x_2} + k_{x_1}^-)k_{y_3}^- + (a_{x_1}a_{x_2} + a_{x_1}k_{x_1}^- + a_{x_2}k_{x_1}^+)a_{y_3} + (a_{x_1}a_{x_2} + a_{x_1}k_{x_1}^- + a_{x_2}k_{x_1}^+)k_{y_3}^+)} \\
[x_3] &= \frac{a_{y_2}k_{x_3}^-\delta' + (a_{y_1}a_{y_2} + (a_{y_2} + k_{y_1}^-)k_{x_3}^- + a_{y_1}k_{y_1}^- + a_{y_2}k_{y_1}^+)\delta}{(a_{x_3}(a_{y_2} + k_{y_1}^-)k_{x_3}^- + (a_{y_1}a_{y_2} + a_{y_1}k_{y_1}^- + a_{y_2}k_{y_1}^+)a_{x_3} + (a_{y_1}a_{y_2} + a_{y_1}k_{y_1}^- + a_{y_2}k_{y_1}^+)k_{x_3}^+)} \\
[y_1] &= \frac{(a_{x_3}a_{y_2}\delta' + (a_{y_2} + k_{y_1}^-)\delta k_{x_3}^+ + a_{y_2}\delta' k_{x_3}^+)}{(a_{x_3}(a_{y_2} + k_{y_1}^-)k_{x_3}^- + (a_{y_1}a_{y_2} + a_{y_1}k_{y_1}^- + a_{y_2}k_{y_1}^+)a_{x_3} + (a_{y_1}a_{y_2} + a_{y_1}k_{y_1}^- + a_{y_2}k_{y_1}^+)k_{x_3}^+)} \\
[y_2] &= \frac{-(a_{x_3}a_{y_1}\delta' + a_{x_3}\delta' k_{x_3}^- + a_{y_1}\delta' k_{x_3}^+ - \delta k_{x_3}^+ k_{y_1}^+)}{(a_{x_3}(a_{y_2} + k_{y_1}^-)k_{x_3}^- + (a_{y_1}a_{y_2} + a_{y_1}k_{y_1}^- + a_{y_2}k_{y_1}^+)a_{x_3} + (a_{y_1}a_{y_2} + a_{y_1}k_{y_1}^- + a_{y_2}k_{y_1}^+)k_{x_3}^+)} \\
[y_3] &= \frac{(a_{x_1}a_{x_2}\delta' + a_{x_1}\delta' k_{x_1}^- + a_{x_2}\delta' k_{x_1}^+ + (a_{x_2}\delta + a_{x_2}\delta' + \delta' k_{x_1}^-)k_{y_3}^-)}{(a_{y_3}(a_{x_2} + k_{x_1}^-)k_{y_3}^- + (a_{x_1}a_{x_2} + a_{x_1}k_{x_1}^- + a_{x_2}k_{x_1}^+)a_{y_3} + (a_{x_1}a_{x_2} + a_{x_1}k_{x_1}^- + a_{x_2}k_{x_1}^+)k_{y_3}^+)}
\end{aligned}$$

Again the condition that the rate constants are all positive (> 0) gives a strict sign to the coefficients of δ and δ' in the expressions of concentrations. We can then replace these expressions in equation (S4.18) and taking care only of the sign, we get two new equations:

$$
\begin{aligned}
0 &= A\delta' - B\delta - C\delta\delta' - D\delta'^2 - E\delta^2 \\
0 &= -A'\delta' + B'\delta - C'\delta\delta' - D'\delta'^2 - E'\delta^2
\end{aligned}
\tag{S4.24}
$$

where A, B, C, D, E and A', B', C', D', E' are functions of the rates, and for positive rate constants, all of them have positive values.

The stationary states of the type II $\ell = 2$ core correspond to the solutions of equation (S4.24) in the positive quadrant.

Lemma S4.6. *There is no degenerate stationary state in the positive quadrant for (S4.24)*

Proof. Assume that (S4.24) has a degenerate stationary state in the positive quadrant (not at origin) satisfying the given inequality, then

The Jacobian Matrix of the system at this point is

$$
J = \begin{bmatrix} -B - 2E\delta - C\delta' & A - 2D\delta' - C\delta \\ B' - 2E'\delta - C'\delta' & -A' - 2D'\delta' - C'\delta \end{bmatrix}
$$

The state being degenerate implies that the columns of this matrix are linearly dependent, so assume there exists μ such that: Column 1 + Column 2 $\times \mu \frac{\delta'}{\delta} = 0$. In terms of expressions, this gives

$$
\begin{aligned}
0 &= -B\delta - 2E\delta^2 - C\delta'\delta + \mu(A\delta' - 2D\delta'^2 - C\delta\delta') \\
0 &= B'\delta - C'\delta\delta' - 2E'\delta^2 + \mu(-A'\delta' - 2D'\delta'^2 - C'\delta\delta')
\end{aligned}
\tag{S4.25}
$$

Using equation (S4.24) and replacing terms, these equations can be written as

$$
\begin{aligned}
0 &= (\mu - 1)B\delta + (\mu - 2)E\delta^2 - \mu D\delta'^2 - C\delta\delta' \\
0 &= (1 - \mu)A'\delta' + (1 - 2\mu)D'\delta'^2 - \mu C'\delta\delta' - E'\delta^2
\end{aligned}
\tag{S4.26}
$$

In the second equation of (S4.26), if $\mu > 1$ all terms are negative and equality is not possible. Thus $\mu < 1$. Also from the first equation of (S4.26), $0 < \mu < 1$ is not possible.

Thus $\mu < 0$.
With a negative μ, there is only one term in each equation of (S4.26) different in sign from the rest. This results in the inequalities

$$|\mu|D\delta'^2 > C\delta\delta' \qquad E'\delta^2 > |\mu|C'\delta'\delta \tag{S4.27}$$

Looking at equations (S4.23), if we take them of the form

$$[x_1] = b_{x_1}\delta + c_{x_1}\delta' \qquad [x_3] = b_{x_3}\delta + c_{x_3}\delta' \qquad [y_1] = b_{y_1}\delta + c_{y_1}\delta' \qquad [y_3] = b_{y_3}\delta + c_{y_3}\delta' \tag{S4.28}$$

$$D = \nu_1^- c_{x_1}c_{x_3}, \quad C = \nu_1^-(c_{x_1}b_{x_3} + b_{x_1}c_{x_3}), \quad E' = \nu_2^- b_{y_1}b_{y_3}, \quad C' = \nu_2^-(c_{y_1}b_{y_3} + b_{y_1}c_{y_3}) \tag{S4.29}$$

with all the constants positive. (S4.27) results in

$$|\mu|\nu_1^- c_{x_1}c_{x_3}\delta' > \nu_1^- c_{x_1}b_{x_3}\delta \qquad \nu_2^- b_{y_1}b_{y_3}\delta > |\mu|\nu_2^- b_{y_1}c_{y_3}\delta'$$

$$|\mu|c_{x_3}\delta' > b_{x_3}\delta \qquad b_{y_3}\delta > |\mu|c_{y_3}\delta' \tag{S4.30}$$

Looking back at (S4.23), $b_{x_3} > c_{x_3}$ and $b_{y_3} < c_{y_3}$. This results in contradicting inequalities with δ, δ' and μ. Such a μ cannot exist.

Lemma S4.7. *For degradation of the form $a' = \{\alpha a_{x_1}, \alpha a_{x_2}, \alpha a_{x_3}, \alpha a_{y_1}, \alpha a_{y_2}, \alpha a_{y_3}\}$, the stationary state at the origin has Jacobian determinant 0 for only one value of $\alpha \in \mathbb{R}^+$*

Proof. Similar to the type IV case, the Jacobian matrix is of the form

$$J = \begin{bmatrix} -B & A \\ B' & -A' \end{bmatrix}$$

Assume fixed reaction constants ($\{k,\nu\}$). $a = \{a_{x_1}, a_{x_2}, a_{x_3}, a_{y_1}, a_{y_2}, a_{y_3}\}$, and let us consider the same system with degradation rates of the form ($a' = \{\alpha a_{x_1}, \alpha a_{x_2}, \alpha a_{x_3}, \alpha a_{y_1}, \alpha a_{y_2}, \alpha a_{y_3}\}$) where $\alpha > 0$.
And using equations (S4.23), the Jacobian determinant can be written as

$$Det\ J = F(\alpha, k, \nu, a) - f(k, \nu)f'(k, \nu) \tag{S4.31}$$

where $F(\alpha, k, \nu, a)$ is, in fact, a degree 5 polynomial in α with all positive coefficients which is a strictly increasing function of α. For fixed rates and varying $\alpha > 0$, $Det\ J = 0$ for only one value of α.

Lemmas 8.6-8 imply that the Type II$_2$ cycle also satisfies the assumptions of Theorem 5.1 in the main text, if the zero stationary state is non-degenerate for vanishing degradation rates.

S4.3 Some degradations zero and some non-zero for Type II and Type IV

The above lemmas still hold if for the species in each equation of (S4.34) and of (S4.6), at least one has non-zero degradation. For Type IV, this corresponds to at least one of the species from $\{x', u, v\}$ and one from $\{x, w\}$ having non-zero degradation. For Type II the corresponding pair of sets are $\{x_3, y_1, y_2\}$ and $\{y_3, x_1, x_2\}$.

For the case where there is no degradation for all the species for one set of the pair, (S4.23) and (S4.11) become invalid. But taking a look at **Lemma 4.3** (main text), we note that these sets correspond to the linkage classes of each of these types without degradation. Thus if only one of these two sets of each type has non-zero degradation for some species but there is no degradation for any species of the other set, the deficiency is still 0 and we only have a unique stationary state. This encompasses all possible degradation sets.

S4.4 Type V

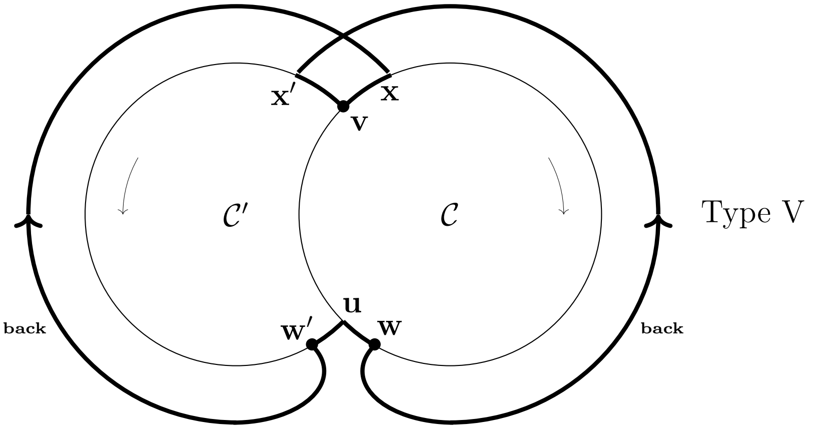

$\mathcal{X} = \{x, x', u, v, w, w'\}$
This time we introduce three variables, δ, δ' and δ''.

$$\delta = \nu_1^+[v] - \nu_1^-[x][x'], \quad \delta' = \nu_2^+[w] - \nu_2^-[u][x'], \quad \delta'' = \nu_3^+[w'] - \nu_3^-[u][x] \qquad \text{(S4.32)}$$

Removing non-fork species, the set of equations are

$$\begin{aligned}
-(k_x^+ + a_x)[x] + \delta + \delta'' + k_x^-[w] &= 0 \\
-(k_{x'}^+ + a_{x'})[x'] + \delta + \delta' + k_{x'}^-[w'] &= 0 \\
-(k_u^+ + a_u)[u] + \delta' + \delta'' + k_u^-[v] &= 0 \\
-(k_u^- + a_v)[v] - \delta + k_u^+[u] &= 0 \\
-(k_x^- + a_w)[w] - \delta' + k_x^+[x] &= 0 \\
-(k_{x'}^- + a_{w'})[w'] - \delta'' + k_{x'}^+[x'] &= 0
\end{aligned} \tag{S4.33}$$

Further, from (S4.33) we also get that

$$\delta' + \delta'' - \delta = a_u[u] + a_v[v] \qquad \delta + \delta'' - \delta' = a_w[w] + a_x[x] \qquad \delta + \delta' - \delta'' = a_{w'}[w'] + a_{x'}[x'] \tag{S4.34}$$

So we are restricted to the positive quadrant in the $\delta, \delta', \delta''$ space.

Lemma S4.8. *For type V core with rate constants and degradation values as in (S4.33), there exists a continuous function $m_b(a,k)$, such that any positive stationary state satisfies $m_b(a,k) \geq \max\{[z], z \in \mathcal{X}\}$*

Proof. The inequalities after putting $\delta, \delta', \delta'' \geq 0$ in (S4.33) are of the form

$$\frac{k_x^+ + a_x}{k_x^-}[x] > [w] \qquad \frac{k_u^+ + a_u}{k_u^-}[u] > [v] \qquad \frac{k_{x'}^+ + a_{x'}}{k_{x'}^-}[x'] > [w'] \tag{S4.35}$$

Further from S4.32 we get

$$\begin{aligned}
&[v] > \frac{\nu_1^-}{\nu_1^+}[x][x'] \implies \frac{k_x^+ + a_x}{k_x^-}\frac{k_u^+ + a_u}{k_u^-}[u] > \frac{\nu_1^-}{\nu_1^+}[w][x'] \\
&[w] > \frac{\nu_2^-}{\nu_2^+}[u][x']
\end{aligned} \tag{S4.36}$$

Which on composing gives an upper bound for $[x']^2$ with degradation terms only in the numerator (with a positive coefficient), and similarly for $[x]$ and $[u]$ which bound everything.

Concentrations in terms of these variables are

$$\begin{aligned}
[x] &= \frac{(a_w + k_x^-)\delta + (a_w + k_x^-)\delta'' - k_x^-\delta'}{a_w a_x + a_x k_x^- + a_w k_x^+} &\qquad [w] &= \frac{k_x^+\delta + k_x^+\delta'' - (k_x^+ + a_x)\delta'}{a_w a_x + a_x k_x^- + a_w k_x^+} \\
[x'] &= \frac{(a_{w'} + k_{x'}^-)\delta + (a_{w'} + k_{x'}^-)\delta' - k_{x'}^-\delta''}{a_{w'} a_{x'} + a_{x'} k_{x'}^- + a_{w'} k_{x'}^+} &\qquad [w'] &= \frac{k_{x'}^+\delta + k_{x'}^+\delta' - (k_{x'}^+ + a_{x'})\delta''}{a_{w'} a_{x'} + a_{x'} k_{x'}^- + a_{w'} k_{x'}^+} \\
[u] &= \frac{(a_v + k_u^-)\delta' + (a_v + k_u^-)\delta'' - k_u^-\delta}{a_v a_u + a_u k_u^- + a_v k_u^+} &\qquad [v] &= \frac{k_u^+\delta' + k_u^+\delta'' - (k_u^+ + a_v)\delta}{a_v a_u + a_u k_u^- + a_v k_u^+}
\end{aligned} \tag{S4.37}$$

The rate constants are all positive, and thus S4.32 gives rise to three equations

$$\begin{aligned}
0 &= -A\delta + B\delta' + C\delta'' - D\delta^2 + E\delta'^2 + F\delta''^2 - G\delta\delta' - H\delta\delta'' - I\delta'\delta'' \\
0 &= A'\delta - B'\delta' + C'\delta'' + D'\delta^2 - E'\delta'^2 + F'\delta''^2 - G'\delta\delta' - H'\delta\delta'' - I'\delta'\delta'' \\
0 &= A''\delta + B''\delta' - C''\delta'' + D''\delta^2 + E''\delta'^2 - F''\delta''^2 - G''\delta\delta' - H''\delta\delta'' - I''\delta'\delta''
\end{aligned} \quad \text{(S4.38)}$$

Lemma S4.9. *There is no degenerate stationary state in the positive quadrant for (S4.38)*

Proof. Assume that (S4.38) has a degenerate stationary state in the positive quadrant (not at origin) satisfying the given inequality, then

The Jacobian Matrix of the system at this point is

$$J = \begin{bmatrix} -A - 2D\delta - G\delta' - H\delta'' & B + 2E\delta' - G\delta - I\delta'' & C + 2F\delta'' - H\delta - I\delta' \\ A' + 2D'\delta - G'\delta' - H'\delta'' & -B' - 2E'\delta' - G'\delta - I'\delta'' & C' + 2F'\delta'' - H'\delta - I'\delta' \\ A'' + 2D''\delta - G''\delta' - H''\delta'' & B'' + 2E''\delta' - G''\delta - I''\delta'' & -C'' - 2F''\delta'' - H''\delta - I''\delta' \end{bmatrix}$$

Without loss of generality (utilising the system symmetry), assume $\delta \leq \delta', \delta''$
For it to be singular, there must exist real numbers of the form $(\mu+1)\frac{\delta'}{\delta}$ and $(\omega+1)\frac{\delta''}{\delta}$ which, on multiplying with Column 2 and 3 respectively (and adding the columns) give 0 to show the linear dependence of the columns of the matrix. Simplifications of these equations yield

$$\begin{aligned}
\mu(B\delta' + 2E\delta'^2 - G\delta\delta' - I\delta''\delta') + \omega(C\delta'' + 2F\delta''^2 - H\delta\delta'' - I\delta'\delta'') &= B\delta' + C\delta'' - A\delta > 0 \\
\mu(-B'\delta' - 2E'\delta'^2 - G'\delta\delta' - I'\delta''\delta') + \omega(C'\delta'' + 2F'\delta''^2 - H'\delta\delta'' - I'\delta'\delta'') &= -B'\delta' + C'\delta'' + A'\delta > 0 \\
\mu(B''\delta' + 2E''\delta'^2 - G''\delta\delta' - I''\delta''\delta') + \omega(-C''\delta'' - 2F''\delta''^2 - H''\delta\delta'' - I''\delta'\delta'') &= B''\delta' - C''\delta'' + A''\delta > 0
\end{aligned} \quad \text{(S4.39)}$$

From S4.37, we also get the relations $B' > C' = A', E' > F', D'$. Take the second equation of S4.38, we get the form

$$C'\delta'' + 2F'\delta''^2 - H'\delta\delta'' - I'\delta'\delta'' = (B'\delta' - A'\delta) + (E'\delta'^2 - D'\delta^2) + G'\delta\delta' + F'\delta''^2 \quad \text{(S4.40)}$$

This expression is thus > 0 and $< B'\delta' + E'\delta'^2 + G'\delta\delta' < B'\delta' + 2E'\delta'^2 + G'\delta\delta' + I'\delta''\delta'$
Thus the second equation of S4.39 gives $\omega > \mu$. A similar procedure for the third equation gives $\omega < \mu$, thus a contradiction.

Lemma S4.10. *For degradation of the form $a' = \{\alpha a_x, \alpha a_{x'}, \alpha a_u, \alpha a_v, \alpha a_w, \alpha a_{w'}\}$, the stationary state at the origin has Jacobian determinant 0 for only one value of $\alpha \in \mathbb{R}^+$*

Proof. The Jacobian matrix is of the form

$$J = \begin{bmatrix} -A & B & C \\ A' & -B' & C' \\ A'' & B'' & -C'' \end{bmatrix}$$

Assume fixed reaction constants ($\{k,\nu\}$). $a = \{a_x, a_{x'}, a_u, a_v, a_w, a_{w'}\}$, and let us consider the same system with degradation rates of the form ($a' = \{\alpha a_x, \alpha a_{x'}, \alpha a_u, \alpha a_v, \alpha a_w, \alpha a_{w'}\}$) where $\alpha > 0$.
From S4.37, we get $C = B = k_1$(constant,$= k_u^+$ in this case) and $A = k_1 + f_1(\alpha)$ where f_1 is an increasing monotonic polynomial in α, and similarly for other rows (with functions f_2 and f_3). The determinant takes the form

$$\begin{aligned} Det\ J &= -(k_1 + f_1)(f_2 f_3 + f_2 k_3 + f_3 k_2) + k_1(2k_2k_3 + k_2 f_3) + k_1(2k_2k_3 + k_3 f_2) \\ &= F(k_1, k_2, k_3) - G(\alpha, k_1, k_2, k_3) \end{aligned} \tag{S4.41}$$

where F is fixed for fixed kinetics and as a sum of products of monotonic polynomials, G is also an increasing monotonic polynomial in α. Thus this value can be zero only for one value of α

Lemmas 8.9-11 imply that the Type V cycle also satisfies the assumptions of Theorem 5.1 if the zero stationary state is non-degenerate for vanishing degradation rates.
